# Everybody Copes: An Interprofessional Workshop on Stress, Coping, and Helping Primary Care Patients Manage Medical Stressors

**DOI:** 10.15766/mep_2374-8265.11300

**Published:** 2023-02-14

**Authors:** Caroline F. Z. Stuhlmann, Hannah Spellman, Daniel J. Coletti

**Affiliations:** 1 Sixth-Year Doctoral Candidate, Department of Psychology, The Graduate Center and Hunter College, City University of New York (CUNY); Research Scientist, Institute for Health Services Research in Dermatology and Nursing (IVDP), University Medical Center Hamburg-Eppendorf (UKE); 2 Third-Year Resident, Pediatric Residency Program, Department of General Pediatrics, Case Western Reserve University/University Hospitals Cleveland Medical Center/Rainbow Babies and Children's Hospital; 3 Director of Behavioral Health Services, Division of General Internal Medicine, Northwell Health; Associate Professor, Departments of Psychiatry and Medicine, Donald and Barbara Zucker School of Medicine at Hofstra/Northwell

**Keywords:** Coping, Mindfulness, Stress, Integrated Behavioral Health, Integrative Medicine, Interprofessional Education, Primary Care, Well-Being/Mental Health

## Abstract

**Introduction:**

The value of psychological principles has become apparent in medical settings, especially with the rise of patient-centered care. We aimed to provide a curriculum informing medical providers about the theoretical basis and clinical utility of the social-cognitive model of stress and coping.

**Methods:**

This workshop was delivered to an interprofessional team of faculty and trainees. Our initial pedagogical approach was to relate the concepts of cognitive appraisals and coping strategies to participants’ own stress responses. We then used didactic presentation and small-group activities to explore ways to promote adaptive coping with patients to improve health outcomes. Learners participated in a mindfulness exercise, conceptualized coping strategies given a hypothetical case scenario, and, in small groups, role-played a patient encounter to construct an effective coping repertoire for the patient. Participants completed a prework self-assessment and workshop evaluation form.

**Results:**

The 2.5-hour workshop had 48 participants from five professions (medicine, education, physician assistant, pharmacology, psychology). We received 35 evaluations (73% response rate). Learners reported increased real-world skills (*M* = 8.0 out of 10) and feeling better prepared for working in interprofessional settings (*M* = 7.6 out of 10). Qualitative feedback suggested that participants recognized the importance of individual differences in coping with stress and felt they could categorize strategies into emotion- or problem-focused coping.

**Discussion:**

This workshop provided participants with basic knowledge about the social-cognitive model of stress and coping and allowed them to practice newly learned skills in a role-play as an interprofessional medical care team.

## Educational Objectives

By the end of this workshop, learners will be able to:

1. Identify and explain the social-cognitive model of stress and coping, particularly the role cognitive appraisals play in generating coping strategies to manage external events perceived by a patient as stressful.
2. Recognize individual differences in cognitive appraisals of stress.
3. Distinguish between different coping strategies and assess their effectiveness across medical and nonmedical contexts.
4. Apply coping skills screening during a role-played patient encounter to support the development of effective patient coping strategies in managing medical stressors.

## Introduction

The importance of enhancing the behavioral health capacities of primary health care teams is underscored by extensive research suggesting that social and emotional concerns are far more likely to present in primary care than in behavioral health treatment settings,^1^ creating what has been termed a de facto mental health system.^2^ There has been increased attention to integrating behavioral health professionals and interventions within patient-centered medical homes, which aim to improve access to care, lower costs, and enhance quality of care through coordinated, interdisciplinary team–based efforts.^3^ Evidence suggests that these integrated behavioral health systems improve clinical outcomes, reduce hospital readmissions, and increase patient satisfaction.^4^ Health care utilization patterns, patient preferences, and these new models of integrated behavioral health incentivize holistic care.^3–5^ Primary care teams that aspire to deep levels of integration must include not only behavioral health clinicians but also an interprofessional team with shared competencies to achieve more uniform models for treating the patient in an integrated manner.^6,7^

While research indicates that between 60% and 80% of primary care office visits concern a stress-related issue,^8^ stress management counseling is actually rarely provided.^9^ Providers may feel inadequately trained to offer this type of support^10^ or mental health providers may feel that their contributions will not be well-received in medical environments.^3^ Although it is important to avoid role confusion in practical settings, shared knowledge and competencies can facilitate communication within an interprofessional team and improve care coordination.^3^ Likewise, recent evidence suggests that medical providers are open to and believe there is a benefit from learning behavioral health principles.^11^ Providing a theoretical foundation to discuss stress with patients might include instruction in ways to apply stress and coping theory grounded in social and cognitive psychology. For example, Lazarus and Folkman's transactional theory of stress and coping posits that an individual's perception of a stressor incorporates cognitive, behavioral, and social-environmental factors to appraise the experience of stress as either harmful or threatening.^12^ This appraisal initiates coping processes. Coping is the affective, cognitive, or behavioral response aimed at reducing the demands of the stressor—whether by managing distress or attempting to solve the problem itself.^12^ Coping strategies are often categorized along dimensional lines, including (1) an approach-oriented or avoidant continuum^13^ or (2) classifying coping strategies as problem-focused or emotion-focused.^12^ Some examples of coping strategies include positive reframing, distraction, seeking social support, and creating an action plan.

In an integrated behavioral health care model that focuses on patient education and supports self-management behaviors,^3^ medical providers trained to apply stress and coping theory have a framework to promote adaptive coping skills. Understanding individual differences in how patients perceive stressors enables learners to triage patient needs and provide tailored coping support. For example, some patients may perceive receiving a medical diagnosis to be highly stressful, while others perceive greater stress from enduring medical procedures. Similarly, understanding dimensional aspects of how patients cope with stress (e.g., approach vs. avoidant, problem- vs. emotion-focused) can help providers refine coping repertoires in ways that are uniquely helpful to the patient.

Primary care-focused training in stress and coping is most appropriately delivered in an interprofessional context. Interprofessional education is a collaborative approach that translates theory and practice across academic professions and encourages feedback to improve instructional models.^14^ Interprofessional education has been deemed a useful but underutilized training tool within integrated models of care.^11^ We developed a curriculum describing ways to integrate psychological principles into interprofessional health care training, as interprofessional teams need skills to help patients manage both the medical and psychological components of chronic conditions. Despite growing recognition of the importance of integrated behavioral health in medical practice, published pedagogical resources are limited.^11^ One curriculum, the Social and Behavioral Sciences PearlsToolbox,^15^ outlines core principles for integrating psychological tools into medical practice, including a brief module concerning coping with stress. We extend the literature by providing a more comprehensive review of stress and coping theory and a template for training in the theory's practical clinical application. Here, we outline the design, administration, and evaluative process for dissemination and replication.

## Methods

### Setting and Participants

We developed this educational workshop for the IMPACcT (Improving Patient Access, Care, and cost through Training) primary care training program.^16,17^ IMPACcT's interprofessional faculty has included behavioral health providers from its inception. As part of the program, four to five workshops are conducted annually and include at least one behavioral health topic each year. Workshop participants included primary care faculty, the project's dedicated patient access coordinator,^18^ and trainees from medicine (internal medicine residents and medical students), physician assistant, and pharmacy training programs. Author Caroline F. Z. Stuhlmann was the current psychology trainee in the IMPACcT program and was asked to lead the development and implementation of this curriculum. The 2.5-hour workshop was presented jointly by the authors and conducted in person in a large conference room. The activities of the workshop were deemed to be quality improvement by our institution's institutional review board.

### Prework (Appendix A)

As prework, attendees completed a 16-item online questionnaire developed for the workshop to assess their own cognitive appraisals and coping strategies for medical and nonmedical stressors. Respondents used a visual analog scale to rate the degree to which they would perceive 12 events as stressful (ranging from *not at all stressful* to *extremely stressful*). Stressors were selected to be relevant to the workshop audience (e.g., commuter traffic, working in health care, final exams). Questions pertaining to coping strategies were derived from a modified version of the Brief COPE.^19^ Four items described a scenario and asked participants to choose between dichotomous coping responses to manage each situation (approach vs. avoidance, problem-focused vs. emotion-focused). The questionnaire was administrated 2 weeks prior to the workshop to allow data to be tabulated and presented during the workshop (described in module 1, below). The purpose of the survey was to demonstrate how the audience tended to perceive and cope with real-life daily stressors. Survey completion was educational rather than evaluative, serving to generate reflection and discussion about variability in stress appraisals and coping responses among the learners. This activity highlighted how perceptions of stressors and preferred coping responses should not be assumed, underscoring the importance of focusing on subjective patient experience.

### Workshop

The workshop was delivered as a series of four modules. The first module presented the conceptual framework for the material. Modules 2 and 3 delved deeper into the concepts through group discussion and exemplary exercises. Module 4 consisted of a skill-building exercise that encouraged application of learned concepts to a role-play medical scenario.

#### Module 1: Theory of Stress Appraisal and Coping (40 minutes, Appendix B)

The first module was an interactive presentation of the foundations of stress and coping theory. We introduced key terms, outlined the classification of coping strategies by dimension (e.g., problem-focused vs. emotion-focused, approach-oriented vs. avoidance), and discussed the function of coping strategies in relation to stressors. Periodically throughout this module, we discussed results from the prework questionnaire. After a brief introduction to stress and cognitive appraisals, we provided examples from our prework survey to illustrate the variability in stress appraisals among participants (slides 9–13). Then, following definitions of different coping strategies, we presented how our participants used different coping strategies by depicting prework results (slides 17–18 and 21–22). We noted group differences in coping styles, such as whether trainees were more or less likely than faculty members to endorse using one coping strategy over another. Future instructors should replace data from our respondents with results from their own prework survey as this personally connects the audience with the didactic material. We facilitated dialogue and discussed reactions to prework responses.

#### Module 2: Problem-Focused Coping Exercise (15 minutes, Appendix B, slide 23)

Next, we facilitated a brainstorming activity with the large group to demonstrate problem-focused coping using a primary care-focused case scenario. The objective of this module was to allow the audience to familiarize themselves with cognitive and behavioral components of problem-focused coping and ways providers could match this type of strategy effectively to a patient health-related problem. The patient scenario involved a 47-year-old male with type 2 diabetes and recent A1C glucose level of 8.9 who said, “I am too overwhelmed to check my blood sugars.” We presented a problem-solving algorithm^20^ and utilized poster-sized paper to document ideas proposed by the audience throughout each stage of the problem-focused coping process. This exercise gave attendees an opportunity to share ideas about how each profession would navigate the clinical case.

#### Module 3: Emotion-Focused Coping Exercise (15 minutes, Appendix C)

We then used a guided group mindfulness exercise to demonstrate a strategy for emotion-focused coping because mindfulness has been linked to increased regulation of emotional response.^21^ We gave each participant a piece of chocolate, and author Hannah Spellman led the entire group through a 5-minute mindfulness exercise focused on the chocolate. The exercise exemplified mindfulness concepts of open observation, focused attention, and nonjudgmental acceptance while increasing awareness of the participants’ thoughts, emotions, and experiences.^21^ After the group exercise, we allotted 10 minutes for participants to debrief how they felt throughout the exercise and afterward and how the focused attention affected their emotions.

#### Module 4: Practical Applications—Examining Effectiveness of Coping for Patients and the Coping Coach Case Vignette (30 minutes, Appendix B)

The final module extended the prior discussion of coping strategies in order to facilitate practical application to supporting patients under stress. After a brief discussion examining the assessment of coping effectiveness and matching strategies to stressors, we presented a coping review protocol (slides 27–30) to help learners work through a discussion of coping efforts with patients and act collaboratively to adjust the patient's coping repertoire.

As the capstone exercise for the workshop, we assigned the attendees into groups of seven to eight for the Coping Coach activity. When possible, we dispersed attendees by professional discipline and experience level to create diverse groups. Handouts were distributed (slides 31–34) for a case vignette describing Jessica, a patient reporting pain. One group member role-played Jessica, with other group members serving as members of Jessica's interprofessional team. The small groups were asked to develop a plan to assess Jessica's cognitive appraisals of her identified stressors, elicit coping strategies the patient had tried to utilize, offer additional strategies that might be effective, and conduct a teach-back to review the strategies and develop a plan for the patient. Instructors circulated the room to observe, provide insightful comments, and engage in small-group discussions. During the final debrief with the larger group, the learners in the role of Jessica were asked to provide feedback to the groups regarding the helpfulness provided by the coping review.

### Evaluation (10 Minutes, Appendix D)

All attendees were asked to complete a brief mixed-methods postworkshop evaluation (consisting of closed- and open-ended questions) assessing their perceptions of the workshop and the degree to which learning objectives had been met. Quantitative evaluation included two 11-point Likert-scale items that assessed increased knowledge, applicability of content in one's own life and in aiding patients, and perceived preparedness to deliver covered material in an interprofessional setting as a result of the workshop. The six open-ended reflective questions asked attendees to provide details about their experience. Comparative analyses were conducted on quantitative feedback questions to determine group differences between medical residents and nonlicensed trainees. Open-ended responses were analyzed using qualitative content thematic analysis.

## Results

Forty-eight participants, all part of the IMPACcT program, attended the workshop. There were 11 faculty members, including ones from the department of internal medicine (*n* = 8), graduate medical education (*n* = 1), physician assistant (*n* = 1), and psychology (*n* = 1); four program coordinators; and 33 trainees. The trainees represented a range of fields and levels of experience, with 15 first- through fourth-year medical students (46%), nine pharmacy students (27%), seven internal medicine residents in their second and third years (21%), and two physician assistant students (6%). We obtained postmeeting evaluations from 35 attendees (a 73% completion rate).

### Quantitative Evaluation Data

Using an 11-point Likert scale (0 = *don't agree at all,* 10 = *completely agree*), medical residents and nonlicensed students indicated increased knowledge of practical skills with means of 8.3 and 7.9 out of 10, respectively. Learners reported that they were better prepared to work in an interprofessional setting (medical residents’ *M* = 7.5, nonlicensed students’ *M* = 7.8).

### Qualitative Evaluation Data

Our thematic analysis grouped similar responses to open-ended items. We provide exemplary quotes for emergent themes in the Table. Common take-home messages shared by participants included the importance of leveraging knowledge about how people cope with stress and how understanding patient stressors could improve care. New skills learned from the workshop included the ability to distinguish between emotion- and problem-focused coping and to support patient coping efforts. Learners indicated that they wanted additional training in tangible methods to support patient coping efforts, help navigating maladaptive coping strategies, and ways to transition within a medical visit to a discussion of stress and coping. Some participants noted that theory was helpful to understand the material, but others had remaining questions about how best to apply the theory. Most feedback was positively focused (e.g., the workshop was perceived as relevant, practical, and helpful); however, some learners requested that future iterations of the workshop provide extended time for processing the coping review during case discussions, with even smaller groups.

**Table.**
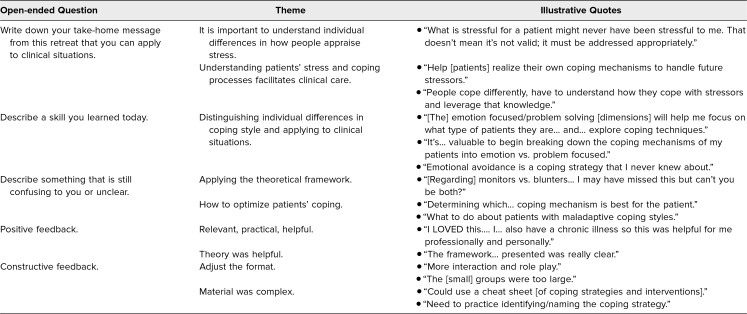
Thematic Review of Qualitative Comments From Participant Evaluations

## Discussion

We designed this workshop to introduce learners to the principles of stress and coping and ways to apply this framework to practical clinical situations. Results of postworkshop evaluations suggested that the workshop succeeded in teaching trainees about new psychological concepts they had not been exposed to previously.

We focused on learners’ self-awareness of their coping responses prior to applying stress and coping constructs to clinical scenarios. Greater understanding of one's own reactions to stress can bolster emotional intelligence, which supports the resilience of providers-in-training as well as enhancing their approach to reducing patient distress.^22^ Furthermore, there is substantial evidence to support the idea that increased stress and inflammation are linked with greater likelihood of onset of chronic illness.^23^ For those living with a chronic illness, poor stress management is associated with poorer outcomes throughout the illness trajectory, affecting prognosis and disease severity.^24,25^ For these reasons, stress reduction and use of effective coping strategies may be key to promoting health for patients in primary care. It should be noted that learning through this workshop does not replace the need for clinical health psychologists, social workers, or other mental health providers, who have advanced skills and training to navigate behavioral health challenges that may co-occur in primary care patients. Rather, we hope that the foundational teachings of stress and coping are useful for all medical providers to better understand the patient experience and enhance the development of patient-centered treatment plans.

Attendees were given the opportunity to share their personal and professional experiences as part of a larger group, which facilitated discussion and interprofessional learning. Through the coping exercises and group activities, attendees were able to integrate the newly learned material with their existing clinical expertise and to role-play helping patients enhance their coping repertoires. Didactic material highlighted individual differences in patient appraisal of stressors and provided examples of coping strategies, emphasizing health conditions and scenarios that could occur in primary care settings. We believe workshops such as ours support efforts to integrate foundational principles of psychology into medical education. Moreover, they offer proof of concept that curricular efforts to teach about the theories, science, and practice of the mind-body connection can instruct with scientific rigor, as well as show practical application.^26^

There were several lessons learned for future delivery of this workshop. The curriculum held the interest of audience members, as evidenced by their reflective questions, stimulating discussion, and active engagement in group activities. Future instructors should consider adding items to prework questionnaires to assess prior workshop knowledge and experience with stress and coping skills in a medical context, which would allow for evaluating within-subject change postworkshop. Despite our limited number of participants, a strength of the workshop was that its small-group activities incorporated voices from several different professional disciplines and allowed greater opportunity for instructors to listen in and engage with each small group. However, time to process questions was important for rich discussion, and in hindsight, we should have allotted more time for module 4, particularly the Coping Coach vignette. Additionally, it was difficult to address whether the groups perceived that they were effective in providing services and psychoeducation to Jessica. Our evaluation questionnaire addressed self-reported knowledge gain and preparedness but not objective skill attainment. Efforts to evaluate how well learners apply the skills learned in the workshop could provide more insight into its effectiveness. One potential method of evaluating the workshop would be for the small groups to self-rate their effectiveness helping Jessica and then compare their ratings to the perceptions of the Jessicas who role-play the patient. In addition, we could have operationalized ratings of positive interactions (e.g., degree to which Jessica perceived that the helpers actively listened, validated patient concerns, provided thoughtful feedback, and tried to incorporate feedback).

This workshop was conducted in person just prior to the COVID pandemic but would also lend itself nicely to a virtual format. Didactic material and large-group exercises could be conducted in a large virtual room, while smaller breakout groups would be better for the Jessica coping review exercise. Participation in the workshop was limited to members of the IMPACcT interprofessional training program, but we envision that the workshop could transition well to larger audiences and include trainees from other professions. In addition, although instructors so far have come from a clinical health psychology background, the materials could be presented by individuals from other behavioral health professions or by anyone acting in a provider role. This workshop serves as a resource for any training program that aims to integrate psychological theory into medical practice, promote interprofessional collaboration, and improve patient-centered care using evidence-based strategies.

## Appendices


Prework.docxSlide Presentation.pptxMindfulness Script.docxEvaluation.docx
*All appendices are peer reviewed as integral parts of the Original Publication.*
