# Supplementary material for: Everybody Copes: An Interprofessional Workshop on Stress, Coping, and Helping Primary Care Patients Manage Medical Stressors
Source: MedEdPORTAL. 2023 Feb 14;19:11300. doi: 10.15766/mep_2374-8265.11300 (PMC9925639; doi:10.15766/mep_2374-8265.11300)
Supplement: Supplementary file 1 — Prework.docxSlide Presentation.pptxMindfulness Script.docxEvaluation.docx [file mep_2374-8265.11300-s001.zip › B. Slide Presentation.pptx]

## Slide 1
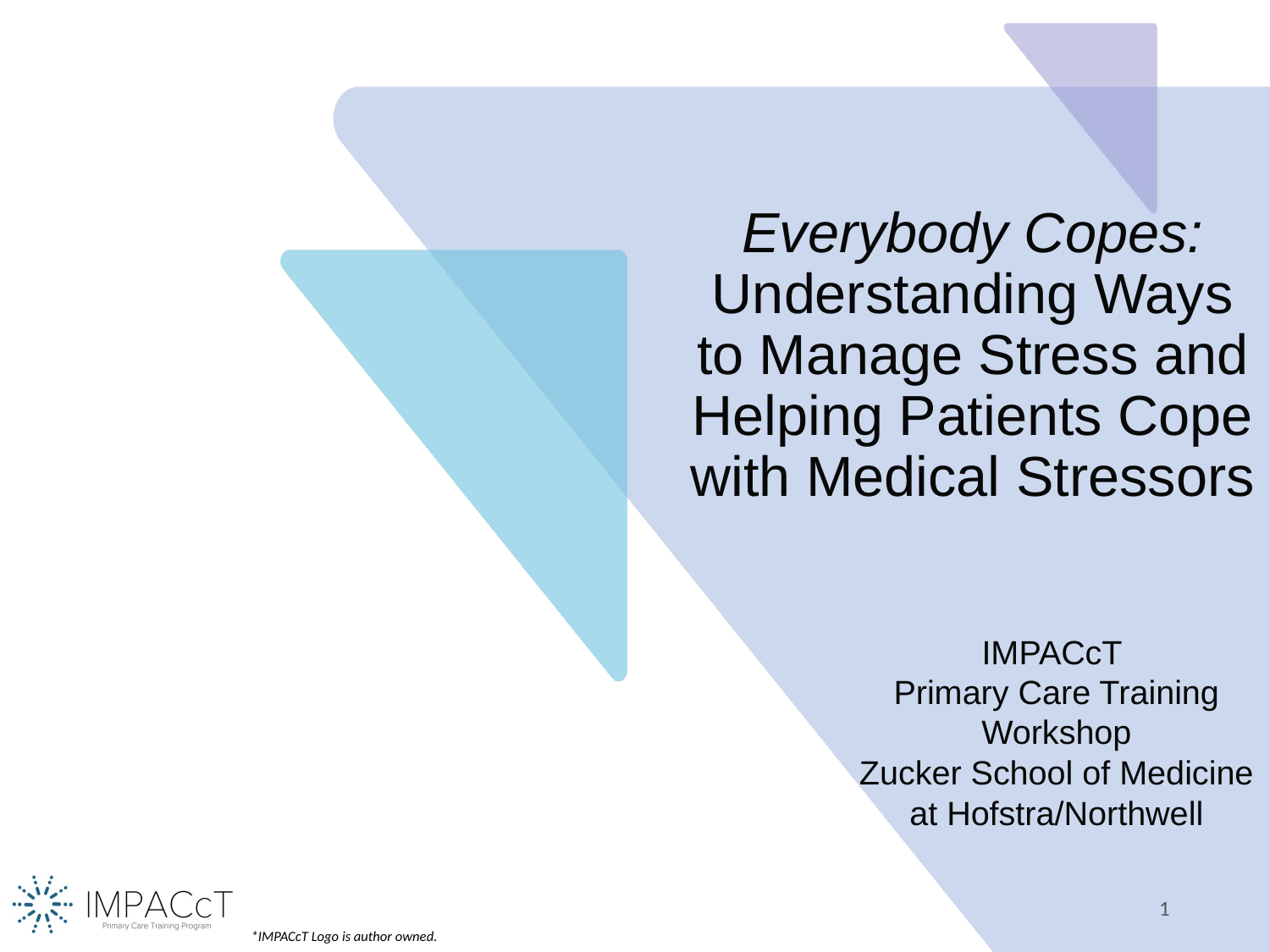

# Everybody Copes: Understanding Ways to Manage Stress and Helping Patients Cope with Medical Stressors
IMPACcT
Primary Care Training Workshop
Zucker School of Medicine at Hofstra/Northwell
1
*IMPACcT Logo is author owned.

## Slide 2
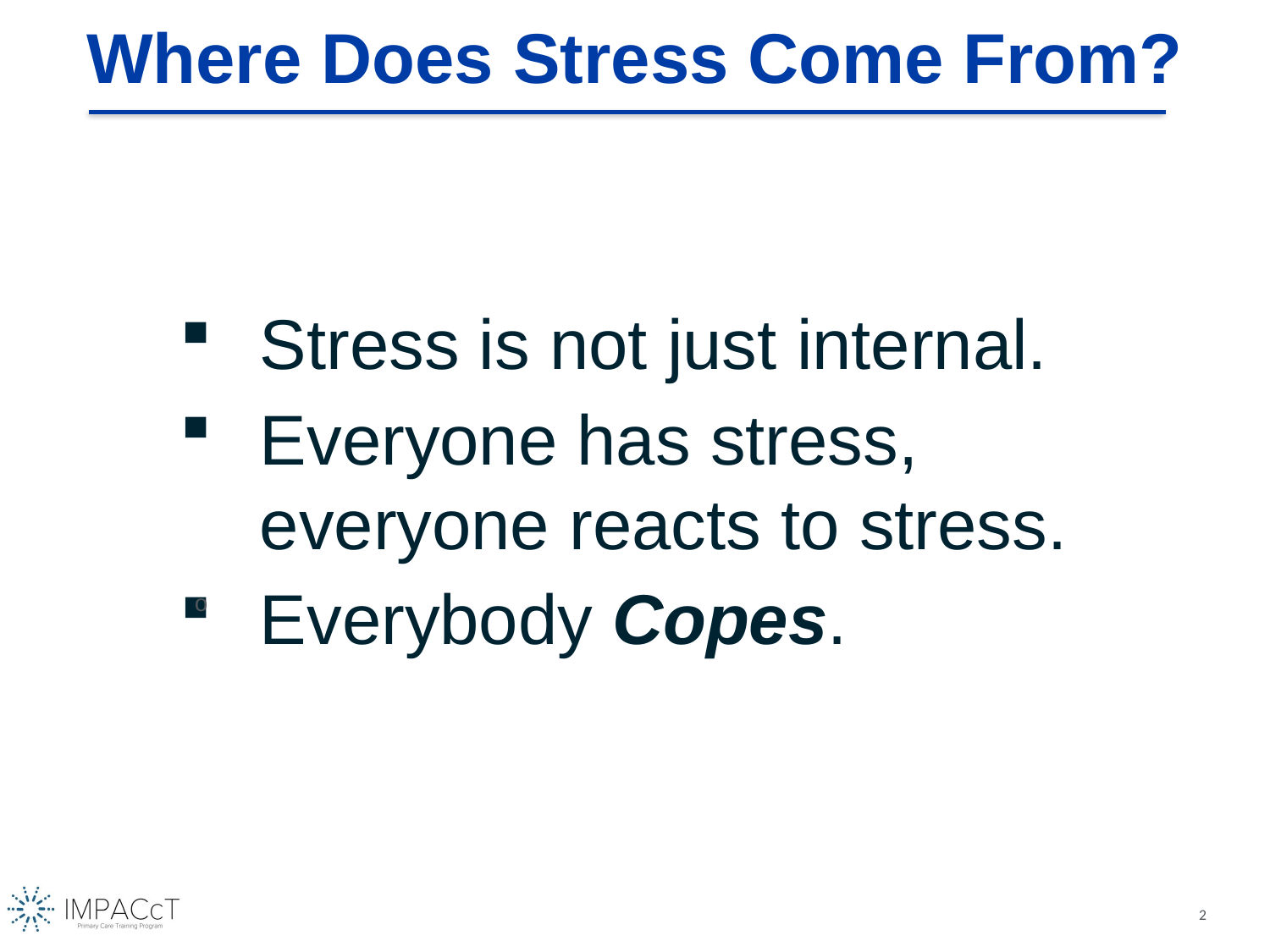

# Where Does Stress Come From?
Stress is not just internal.
Everyone has stress, everyone reacts to stress.
Everybody Copes.
2

## Slide 3
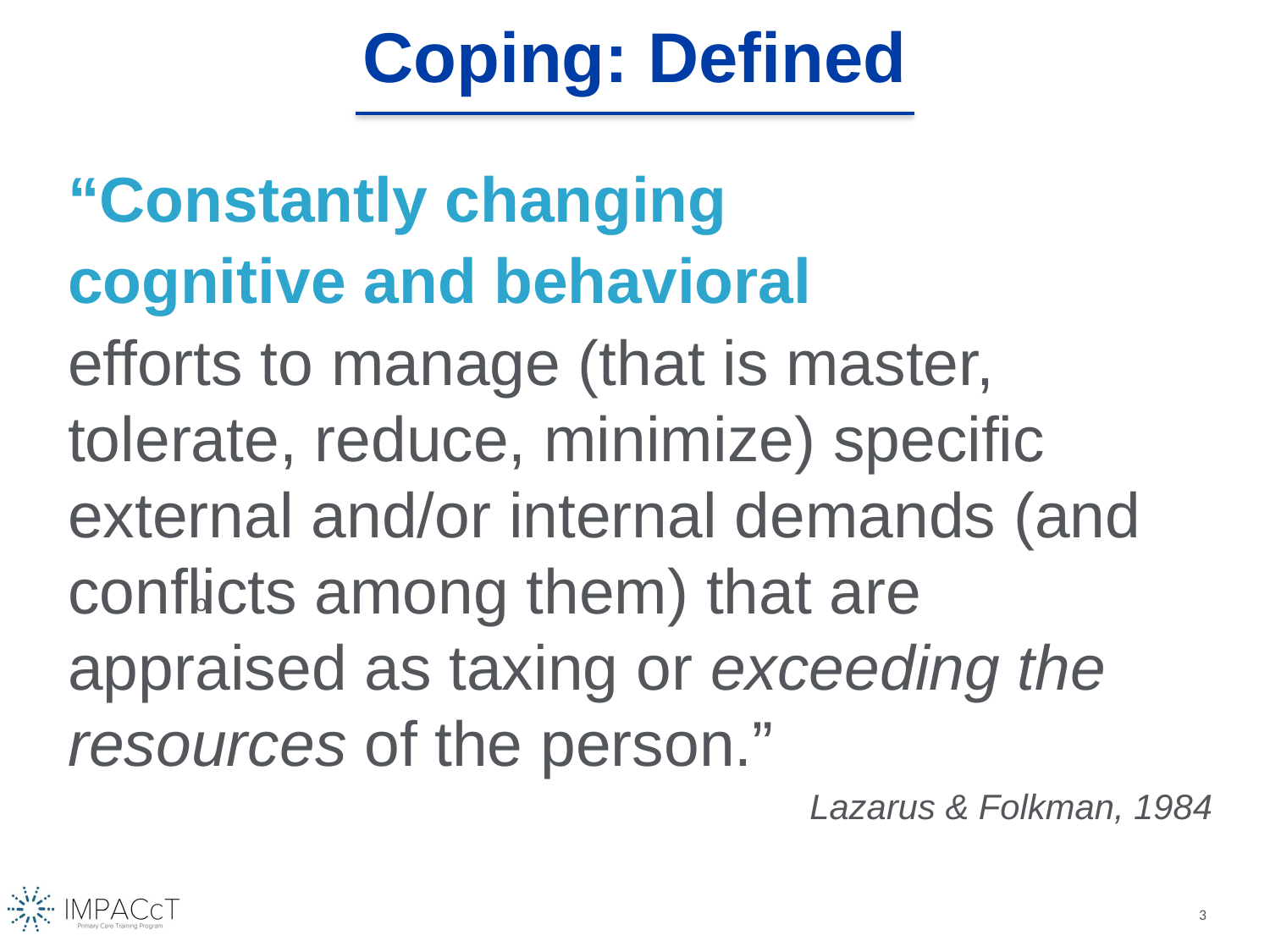

Coping: Defined
“Constantly changing
cognitive and behavioral
efforts to manage (that is master, tolerate, reduce, minimize) specific external and/or internal demands (and conflicts among them) that are appraised as taxing or exceeding the resources of the person.”
Lazarus & Folkman, 1984
3

## Slide 4
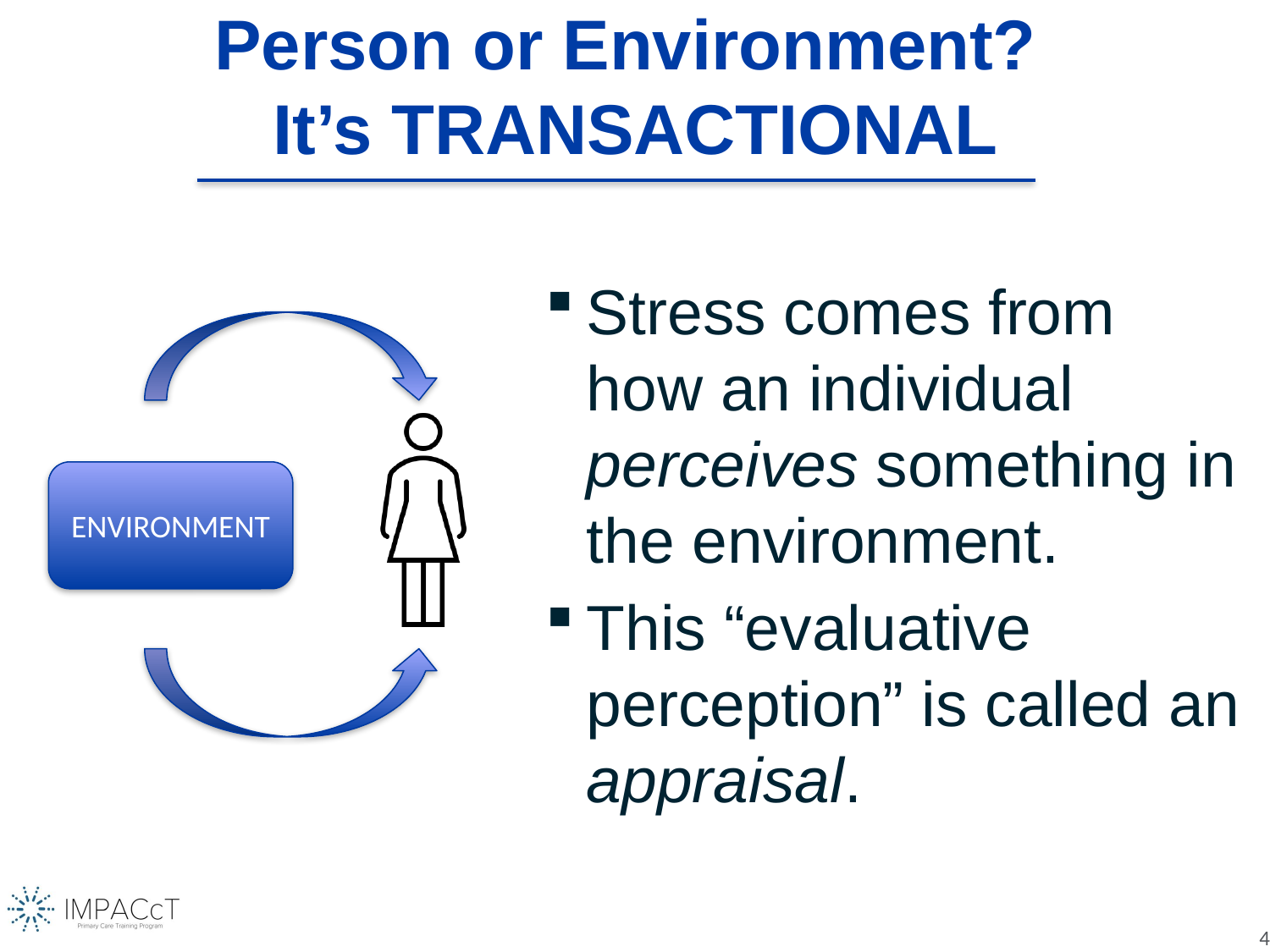

Person or Environment?
It’s TRANSACTIONAL
Stress comes from how an individual perceives something in the environment.
This “evaluative perception” is called an appraisal.
ENVIRONMENT
4

## Slide 5
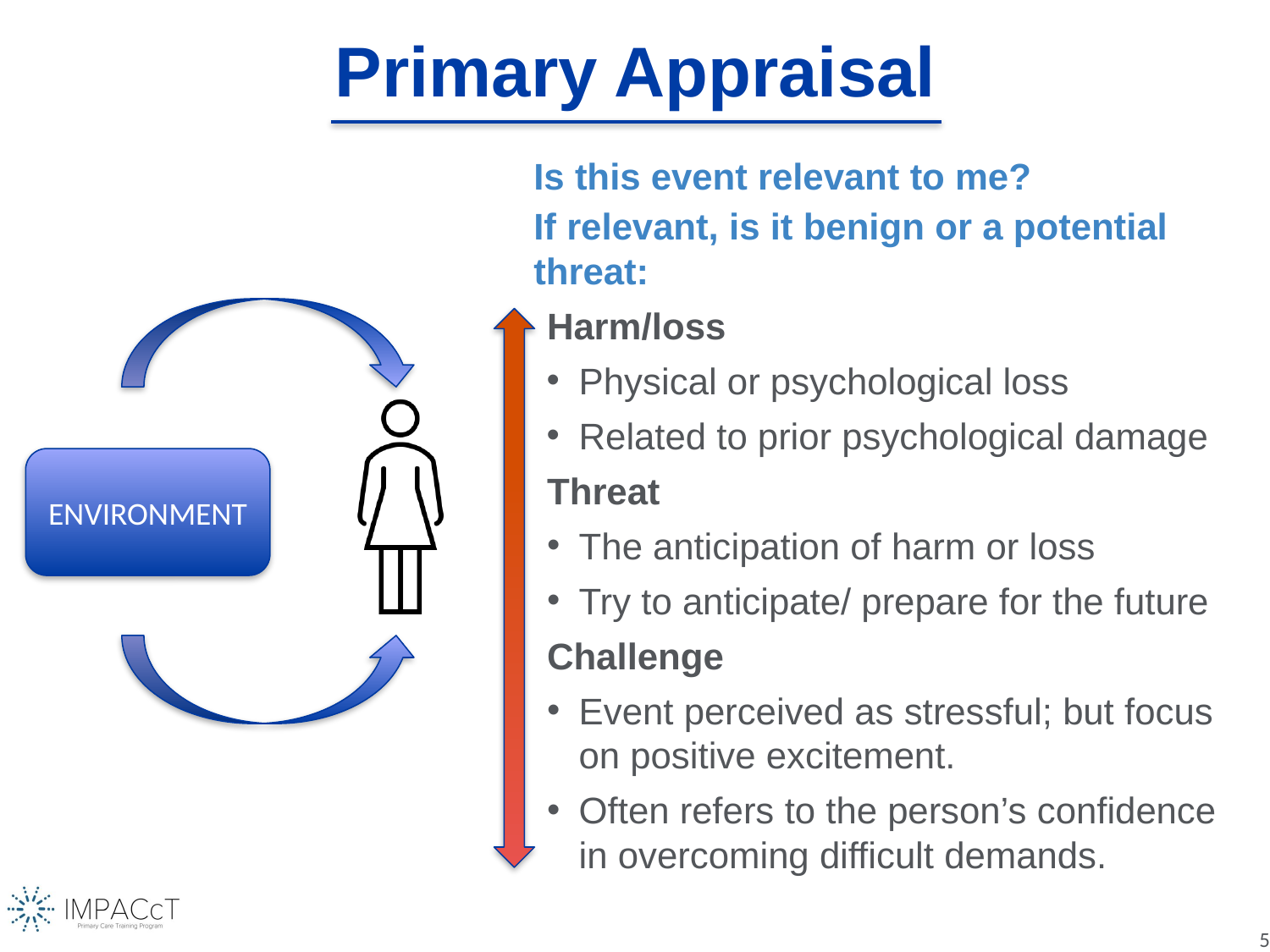

Primary Appraisal
Is this event relevant to me?
If relevant, is it benign or a potential threat:
Harm/loss
Physical or psychological loss
Related to prior psychological damage
Threat
The anticipation of harm or loss
Try to anticipate/ prepare for the future
Challenge
Event perceived as stressful; but focus on positive excitement.
Often refers to the person’s confidence in overcoming difficult demands.
ENVIRONMENT
5

## Slide 6
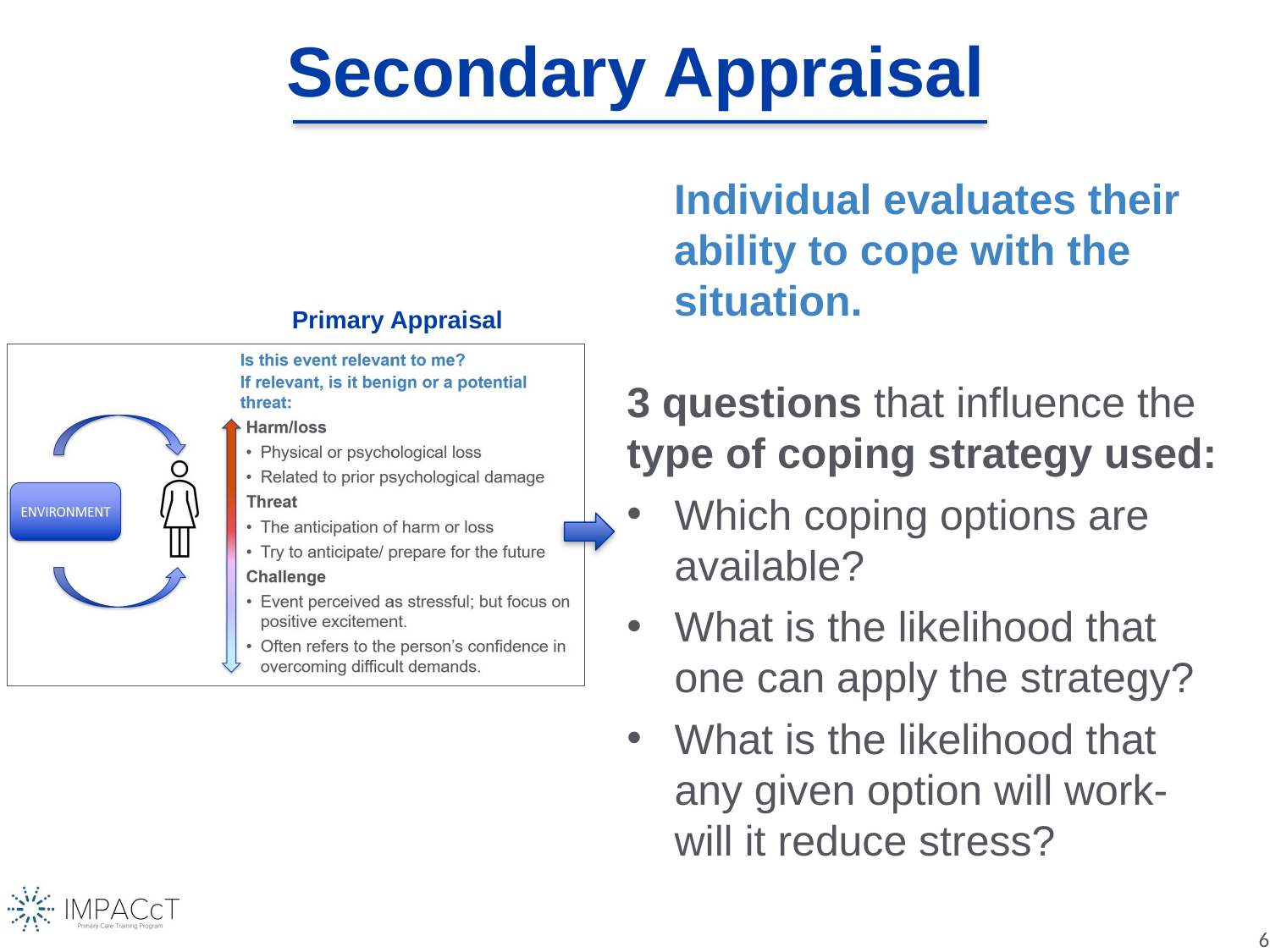

Secondary Appraisal
Individual evaluates their ability to cope with the situation.
3 questions that influence the type of coping strategy used:
Which coping options are available?
What is the likelihood that one can apply the strategy?
What is the likelihood that any given option will work- will it reduce stress?
Primary Appraisal
6

## Slide 7
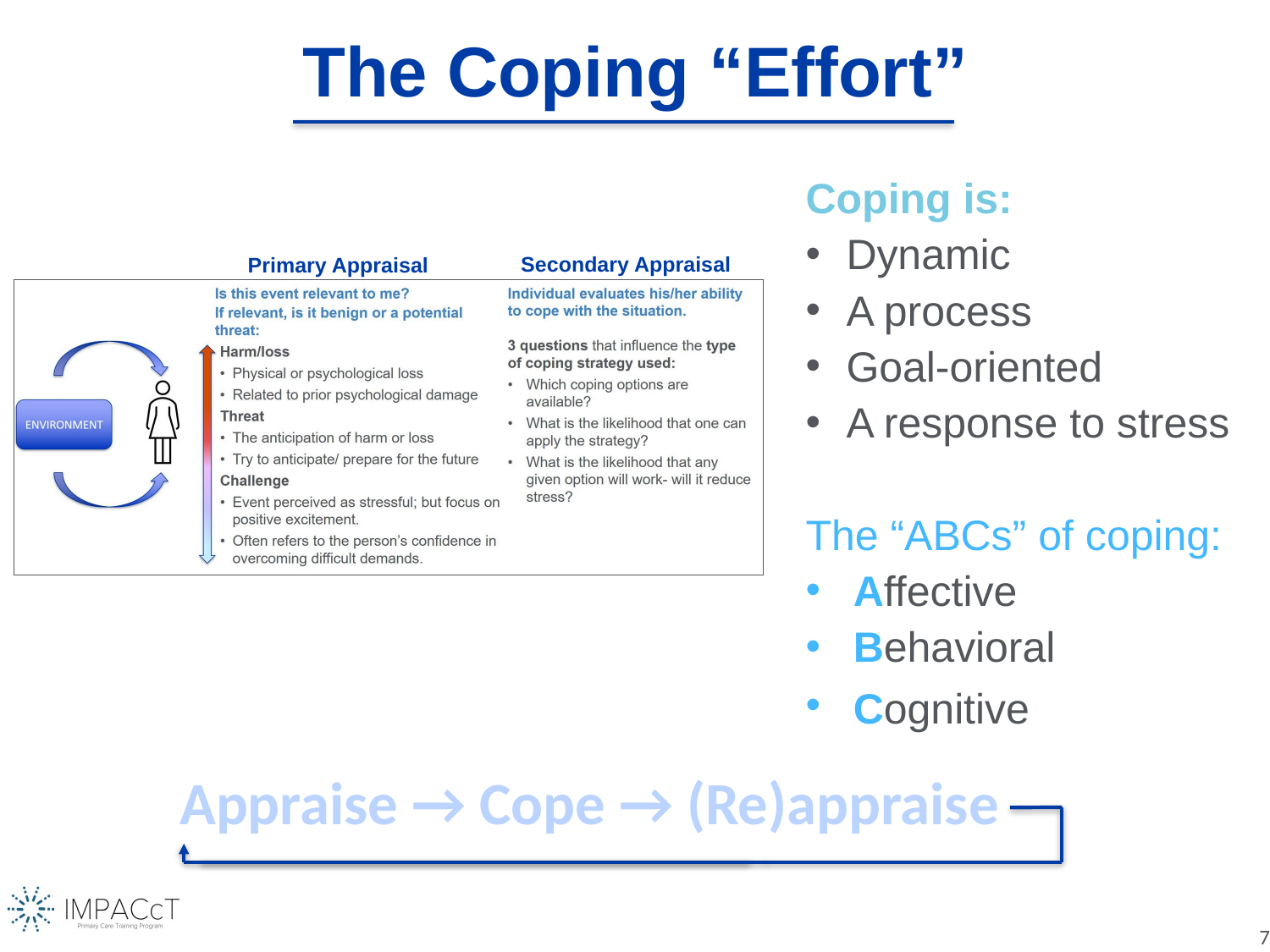

The Coping “Effort”
Coping is:
Dynamic
A process
Goal-oriented
A response to stress
The “ABCs” of coping:
Affective
Behavioral
Cognitive
Secondary Appraisal
Primary Appraisal
Appraise → Cope → (Re)appraise
7

## Slide 8
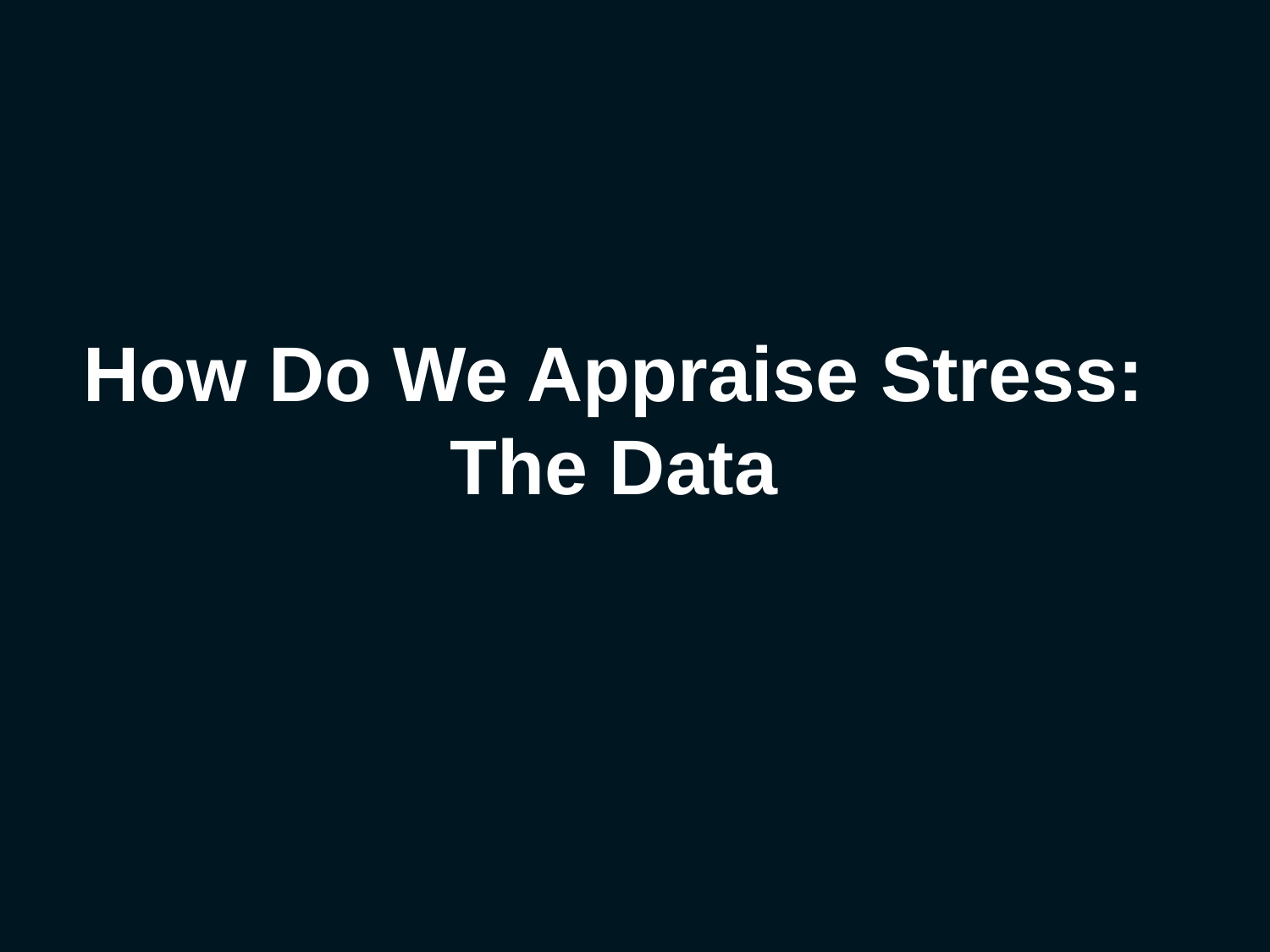

# How Do We Appraise Stress: The Data
8

## Slide 9
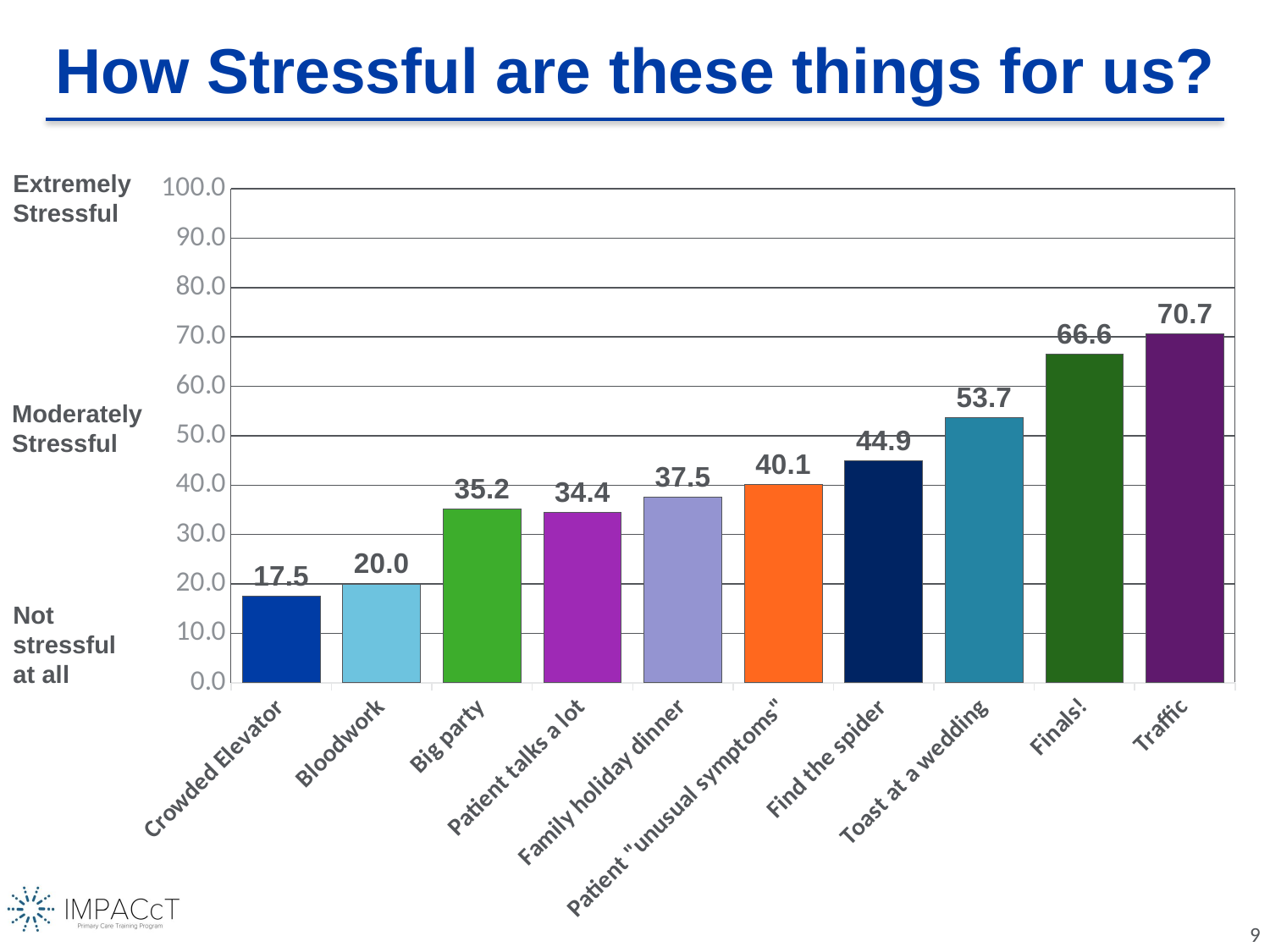

How Stressful are these things for us?
Extremely Stressful
### Chart
| Category | Series 1 |
|---|---|
| Crowded Elevator | 17.51 |
| Bloodwork | 20.0 |
| Big party | 35.15 |
| Patient talks a lot | 34.44 |
| Family holiday dinner | 37.49 |
| Patient "unusual symptoms" | 40.09 |
| Find the spider | 44.86 |
| Toast at a wedding | 53.65 |
| Finals! | 66.56 |
| Traffic | 70.66 |Moderately Stressful
Not stressful
at all
9

## Slide 10
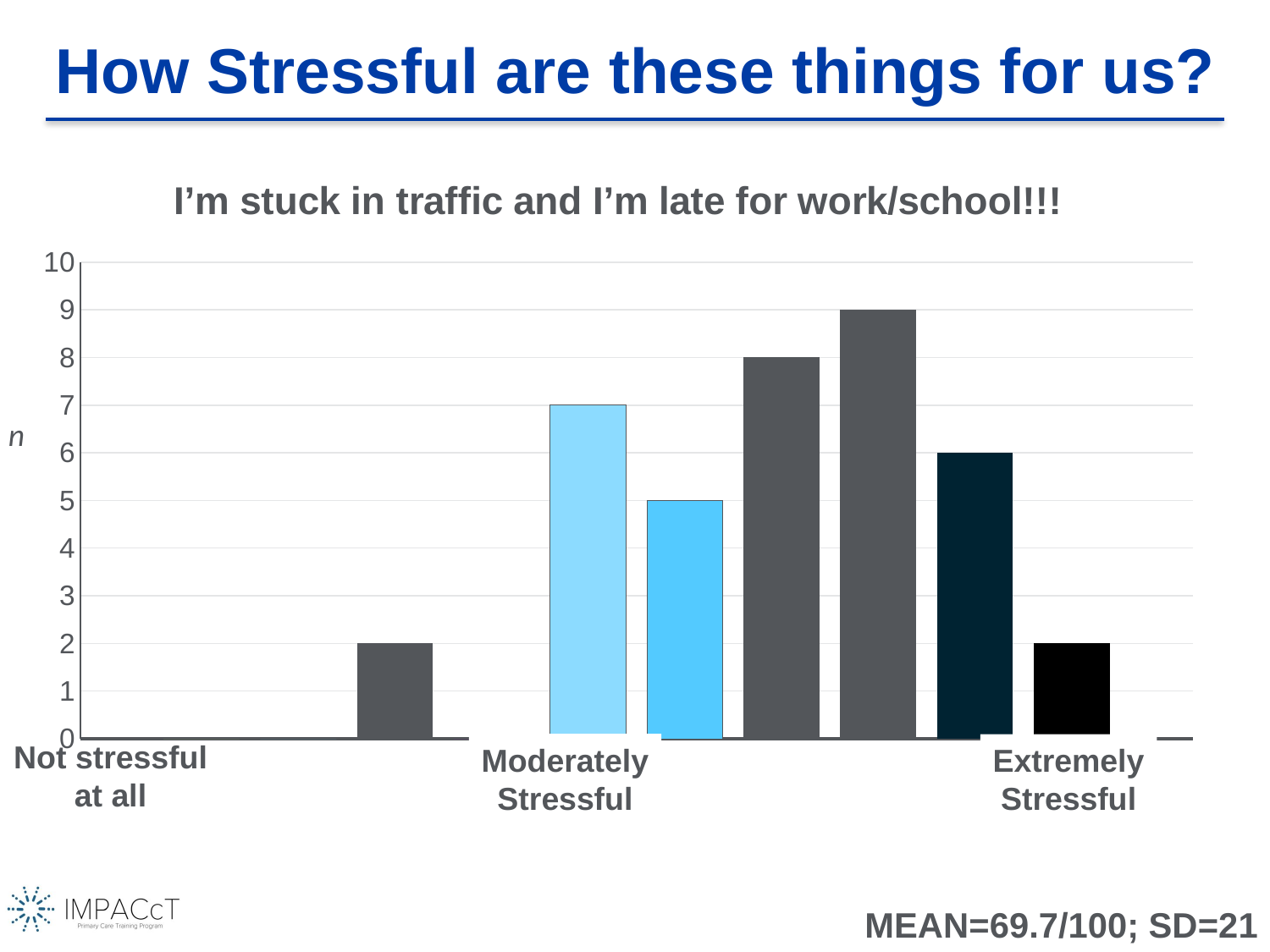

How Stressful are these things for us?
### Chart: I’m stuck in traffic and I’m late for work/school!!!
| Category | 1 | 2 | 3 | 4 | 5 | 6 | 7 | 8 | 9 | 10 |
|---|---|---|---|---|---|---|---|---|---|---|
| 1 | 0.0 | 0.0 | 2.0 | 0.0 | 7.0 | 5.0 | 8.0 | 9.0 | 6.0 | 2.0 |n
Not stressful
at all
Moderately
Stressful
Extremely
Stressful
MEAN=69.7/100; SD=21
10

## Slide 11
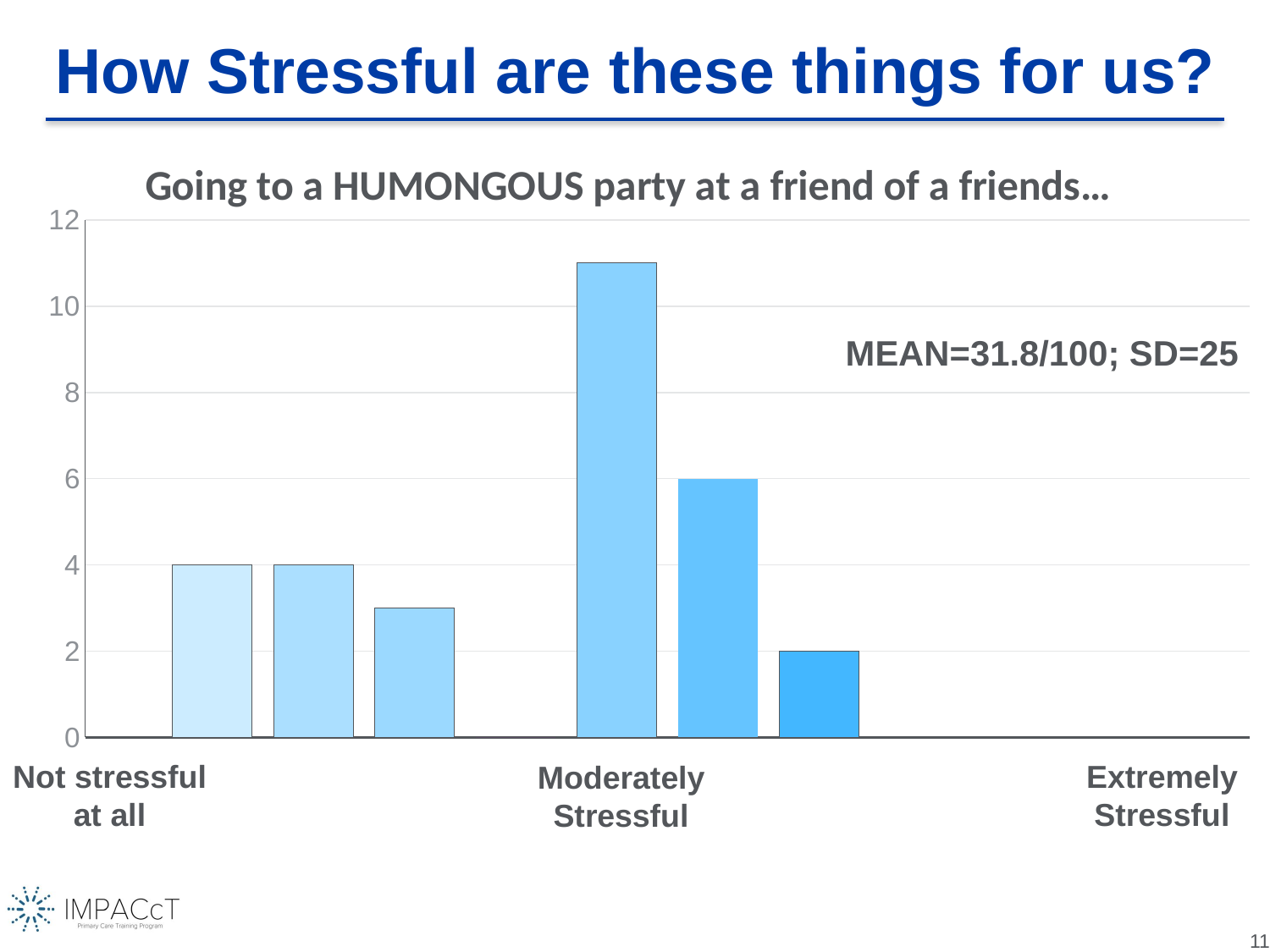

How Stressful are these things for us?
### Chart: Going to a HUMONGOUS party at a friend of a friends…
| Category | 1 | 2 | 3 | 4 | 5 | 6 | 7 | 8 | 9 | 10 |
|---|---|---|---|---|---|---|---|---|---|---|
| 7 | 4.0 | 4.0 | 3.0 | 0.0 | 11.0 | 6.0 | 2.0 | None | None | None |MEAN=31.8/100; SD=25
Not stressful
at all
Extremely
Stressful
Moderately
Stressful
11

## Slide 12
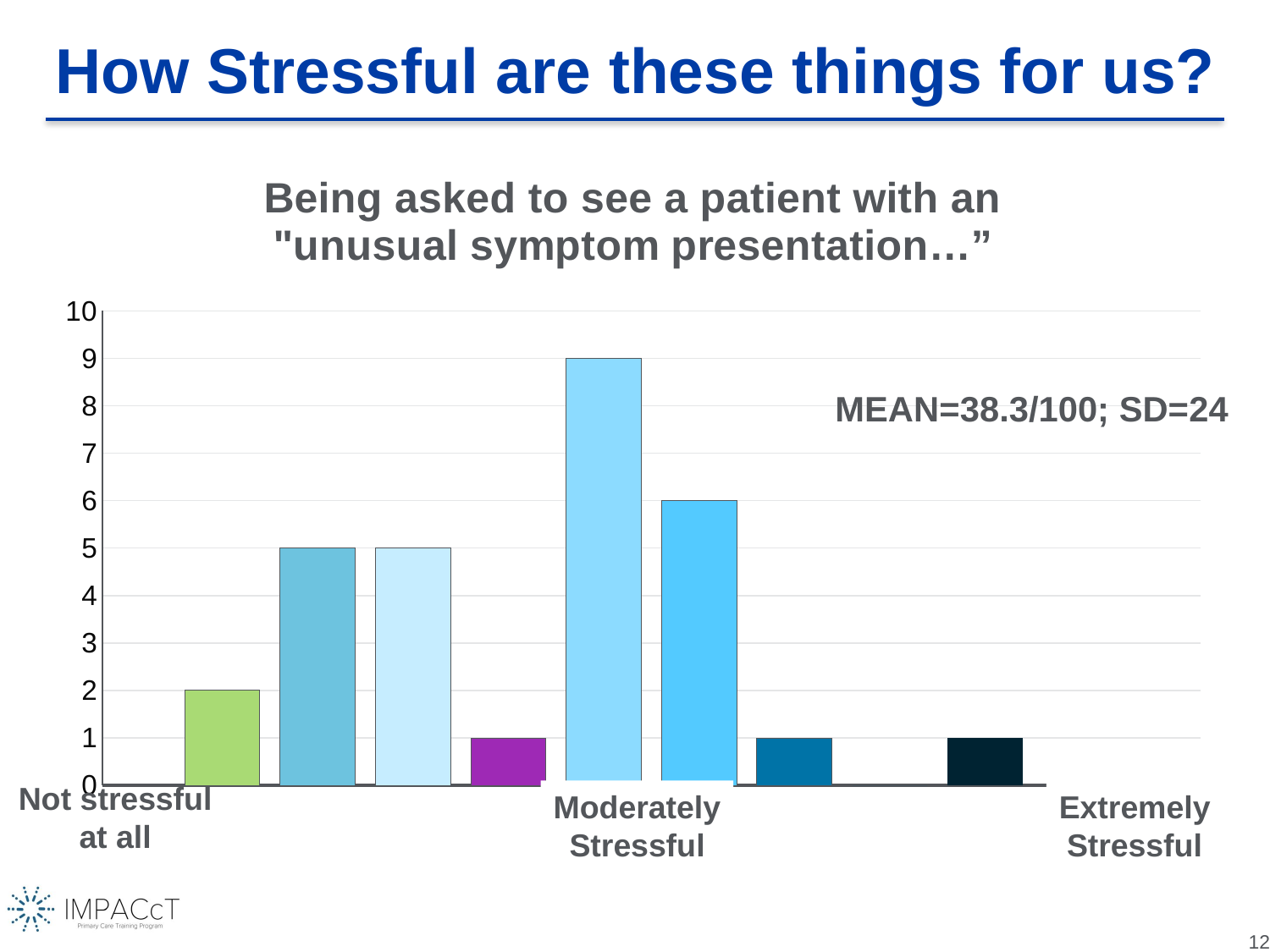

How Stressful are these things for us?
### Chart: Being asked to see a patient with an "unusual symptom presentation…”
| Category | 1 | 2 | 3 | 4 | 5 | 6 | 7 | 8 | 9 | 10 |
|---|---|---|---|---|---|---|---|---|---|---|
| 5 | 2.0 | 5.0 | 5.0 | 1.0 | 9.0 | 6.0 | 1.0 | 0.0 | 1.0 | None |MEAN=38.3/100; SD=24
Not stressful
at all
Moderately
Stressful
Extremely
Stressful
12

## Slide 13
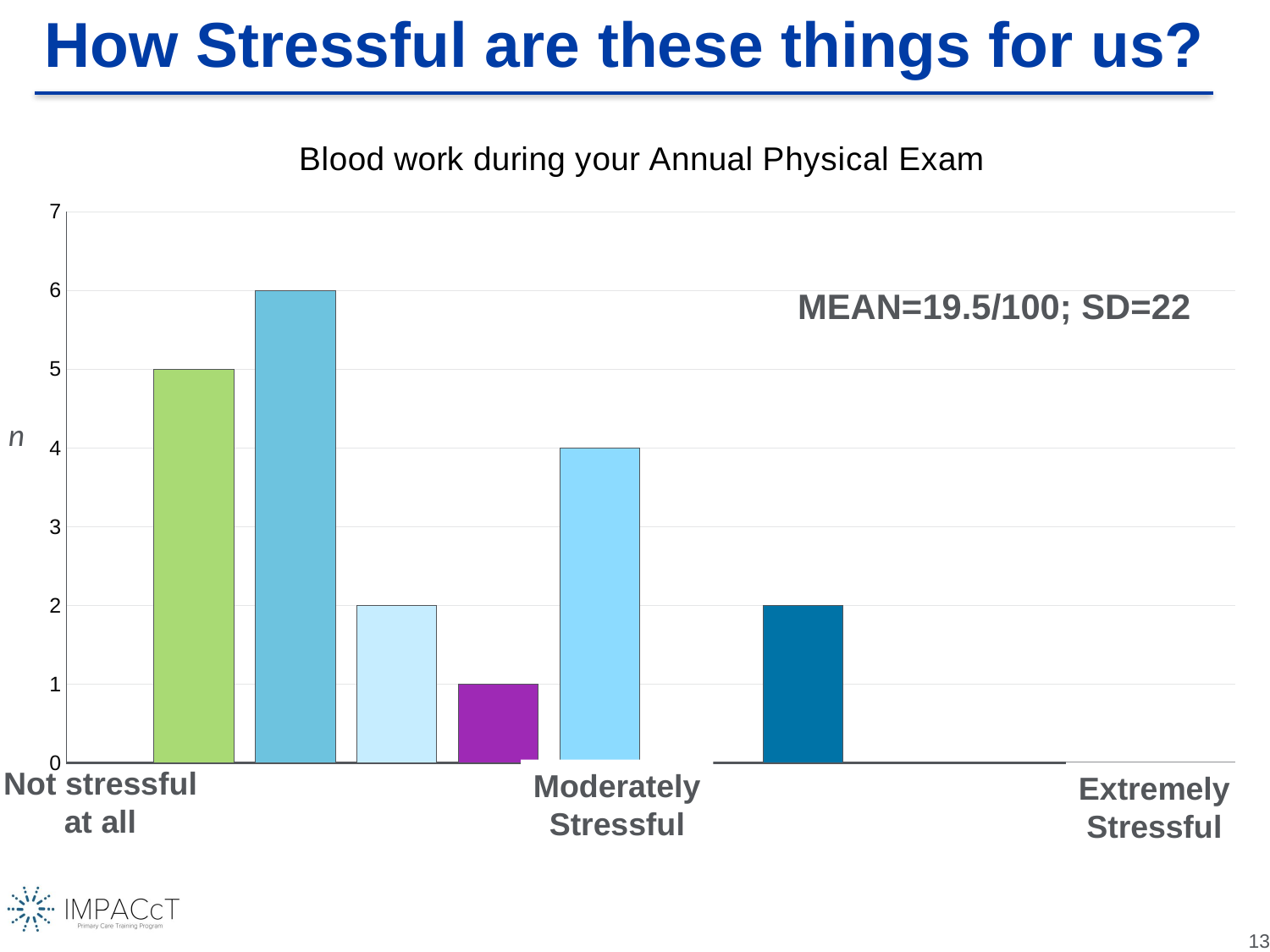

How Stressful are these things for us?
### Chart: Blood work during your Annual Physical Exam
| Category | Column1 | Column2 | Column3 | Column4 | Column5 | Column6 | Column7 | Column8 | Column9 | Column10 |
|---|---|---|---|---|---|---|---|---|---|---|
| 16 | 5.0 | 6.0 | 2.0 | 1.0 | 4.0 | None | 2.0 | None | None | None |MEAN=19.5/100; SD=22
n
Not stressful
at all
Moderately
Stressful
Extremely
Stressful
13

## Slide 14
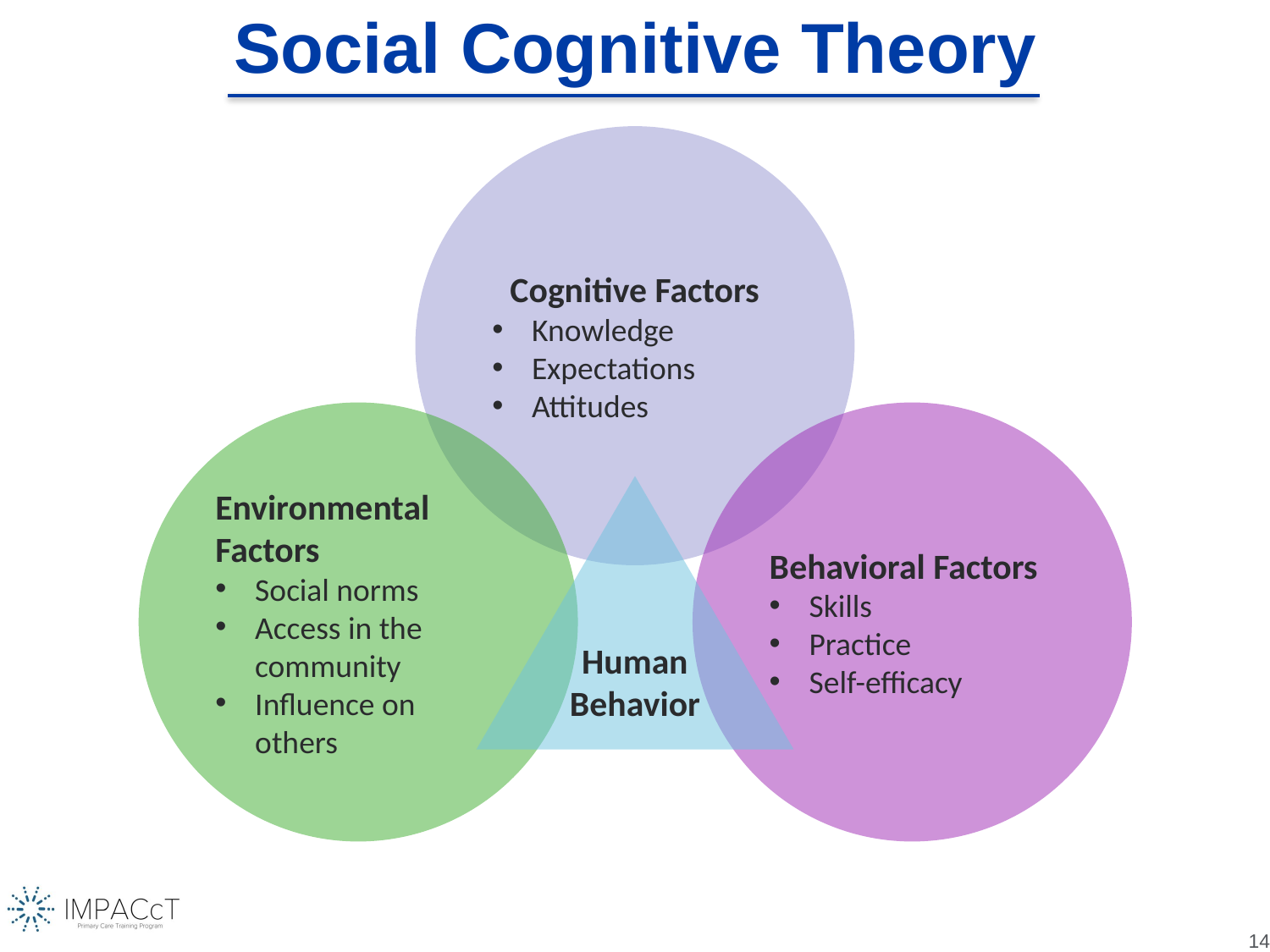

# Social Cognitive Theory
Cognitive Factors
Knowledge
Expectations
Attitudes
Behavioral Factors
Skills
Practice
Self-efficacy
Environmental Factors
Social norms
Access in the community
Influence on others
Human Behavior
14

## Slide 15
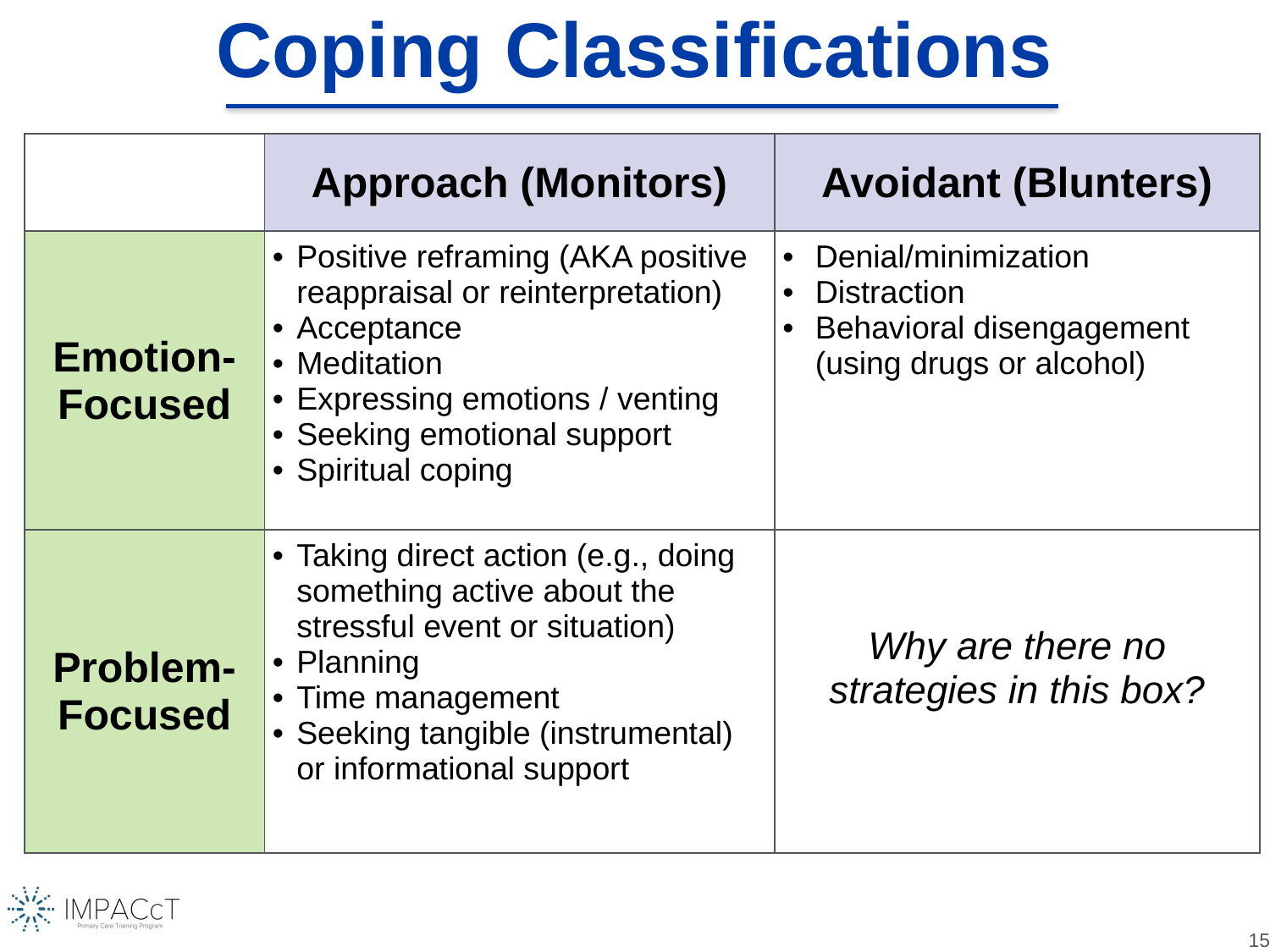

# Coping Classifications
| | Approach (Monitors) | Avoidant (Blunters) |
| --- | --- | --- |
| Emotion-Focused | Positive reframing (AKA positive reappraisal or reinterpretation) Acceptance Meditation Expressing emotions / venting Seeking emotional support Spiritual coping | Denial/minimization  Distraction Behavioral disengagement (using drugs or alcohol) |
| Problem-Focused | Taking direct action (e.g., doing something active about the stressful event or situation) Planning Time management Seeking tangible (instrumental) or informational support | Why are there no strategies in this box? |
15

## Slide 16
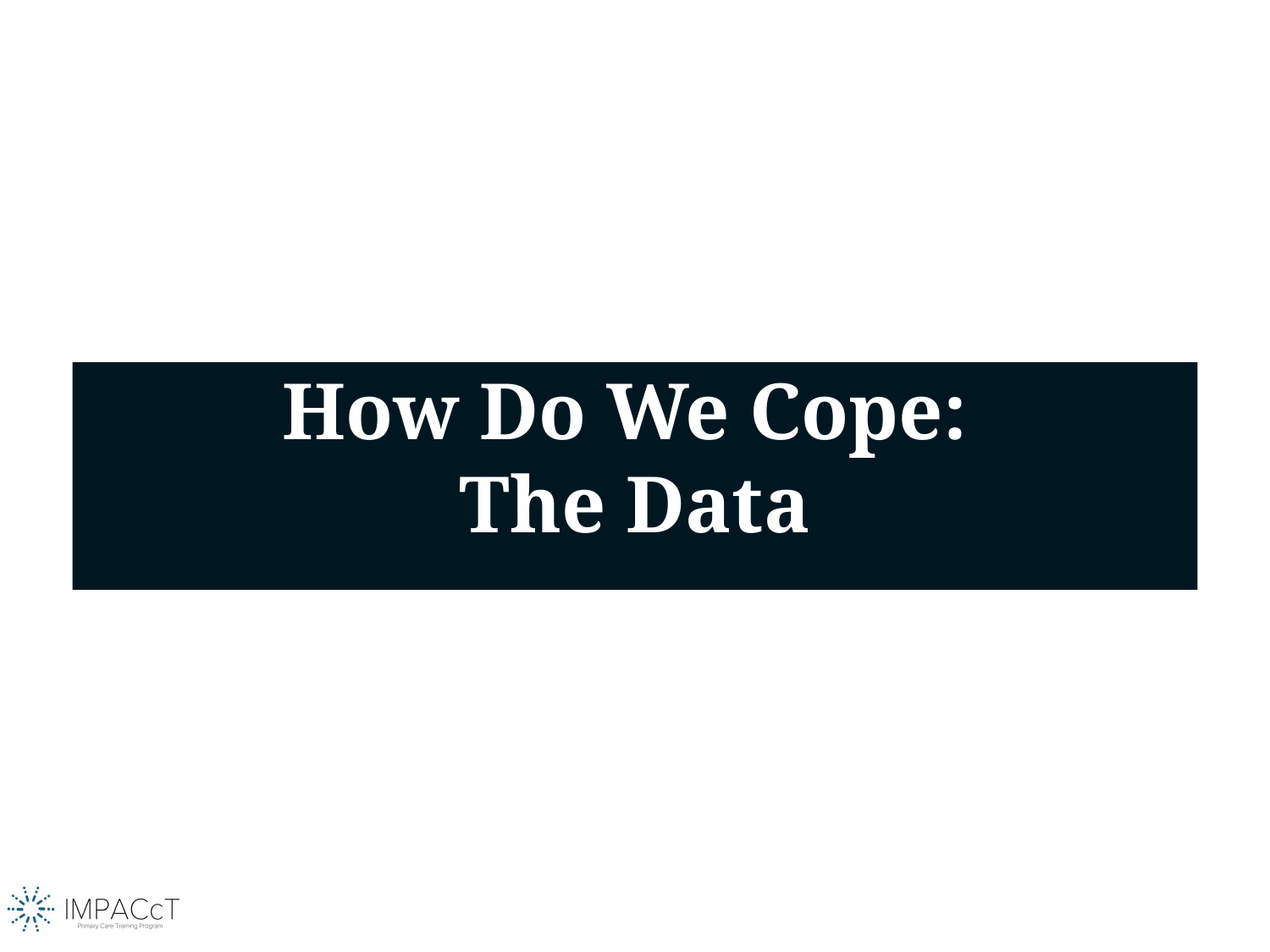

# How Do We Cope: The Data
16

## Slide 17
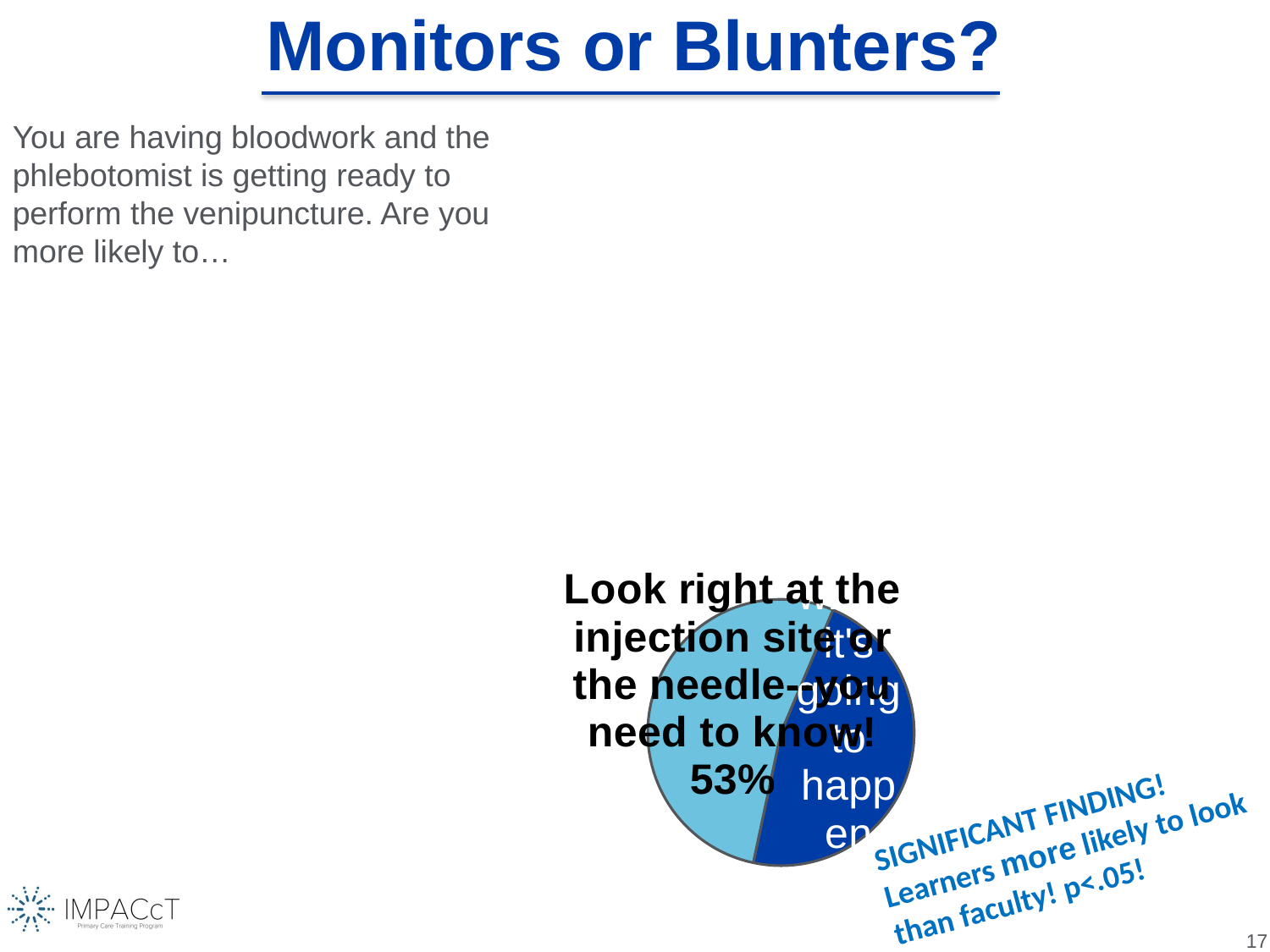

# Monitors or Blunters?
You are having bloodwork and the phlebotomist is getting ready to perform the venipuncture. Are you more likely to…
### Chart
| Category | Sales |
|---|---|
| Look away- I DON'T want to know when it's going to happen | 23.0 |
| Look right at the injection site or the needle--you need to know! | 26.0 |SIGNIFICANT FINDING!
Learners more likely to look than faculty! p<.05!
17

## Slide 18
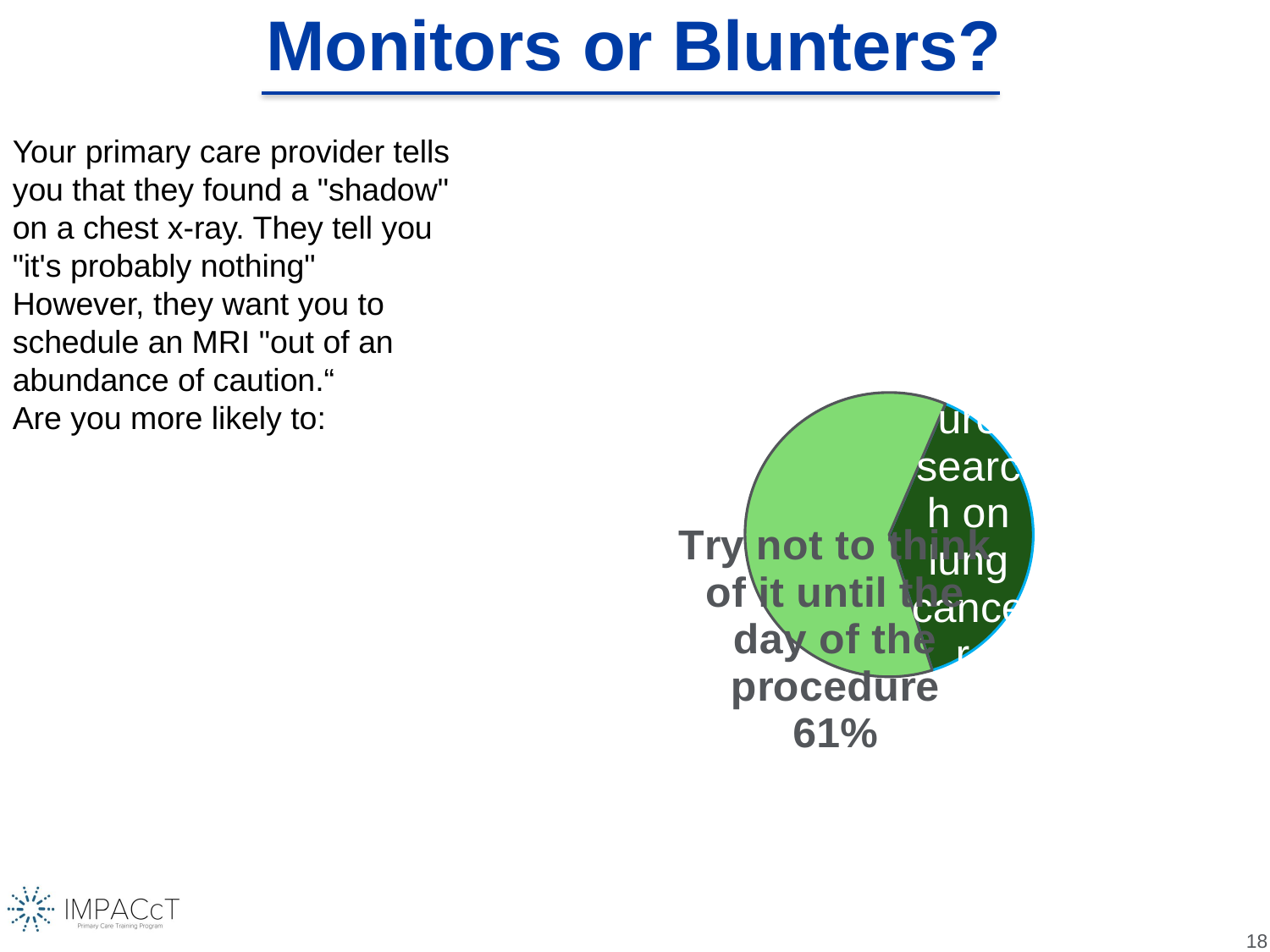

# Monitors or Blunters?
Your primary care provider tells you that they found a "shadow" on a chest x-ray. They tell you "it's probably nothing" However, they want you to schedule an MRI "out of an abundance of caution.“
Are you more likely to:
### Chart
| Category | Sales |
|---|---|
| Perform a PubMed literature search on lung cancer, google like crazy… | 19.0 |
| Try not to think of it until the day of the procedure | 30.0 |18

## Slide 19
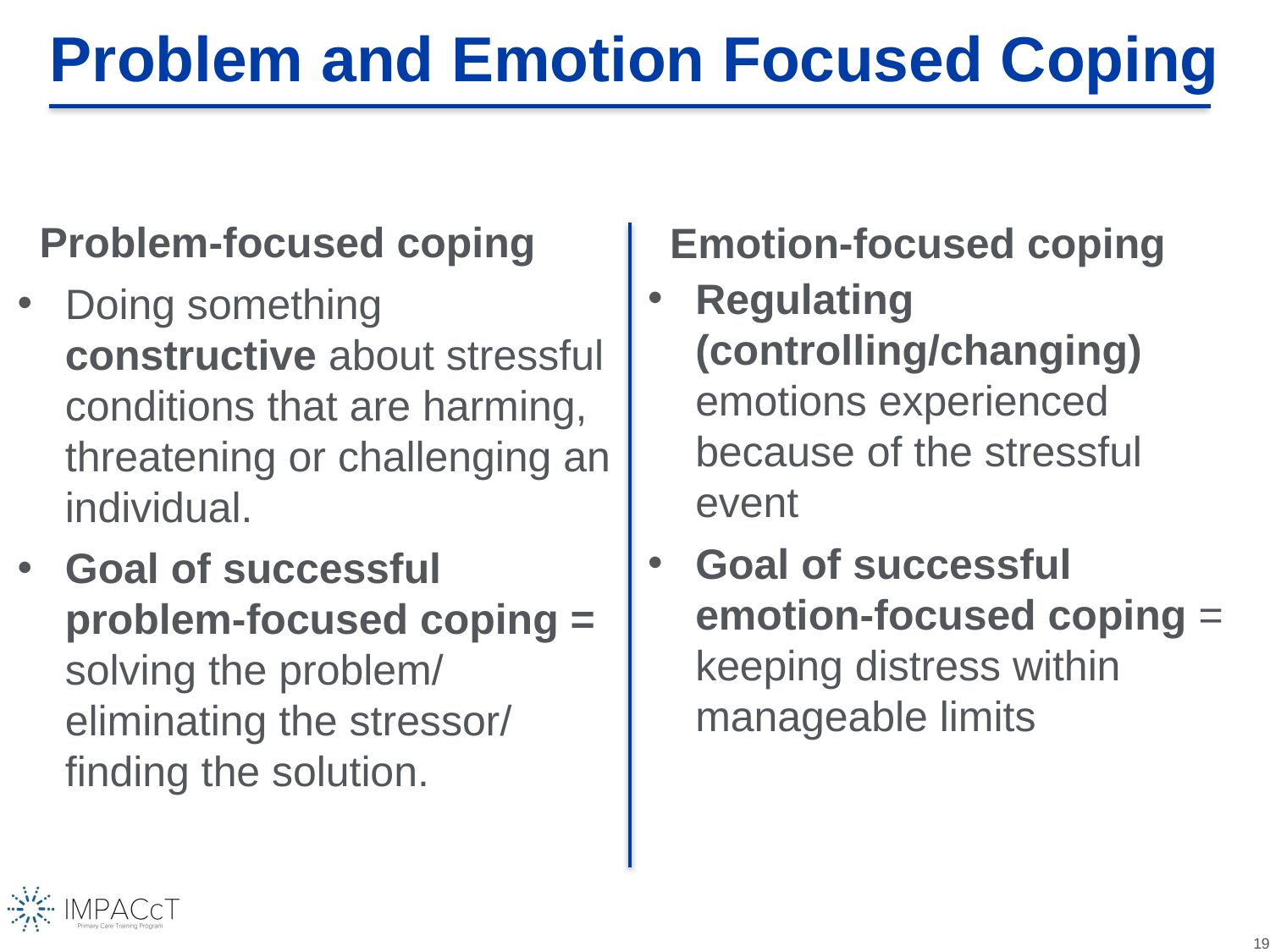

# Problem and Emotion Focused Coping
Problem-focused coping
Doing something constructive about stressful conditions that are harming, threatening or challenging an individual.
Goal of successful problem-focused coping = solving the problem/ eliminating the stressor/ finding the solution.
Emotion-focused coping
Regulating (controlling/changing) emotions experienced because of the stressful event
Goal of successful emotion-focused coping = keeping distress within manageable limits
19

## Slide 20
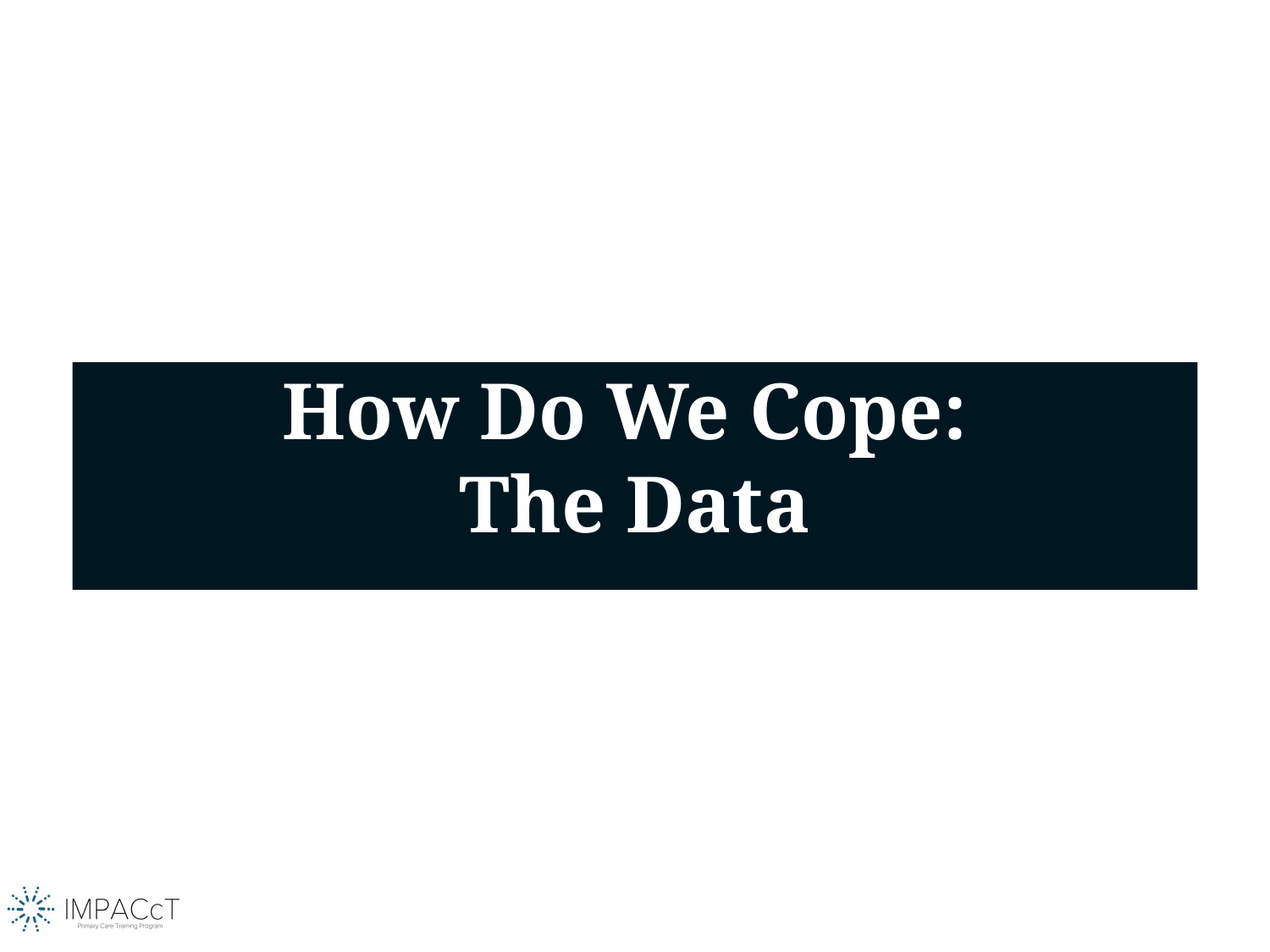

# How Do We Cope: The Data
20

## Slide 21
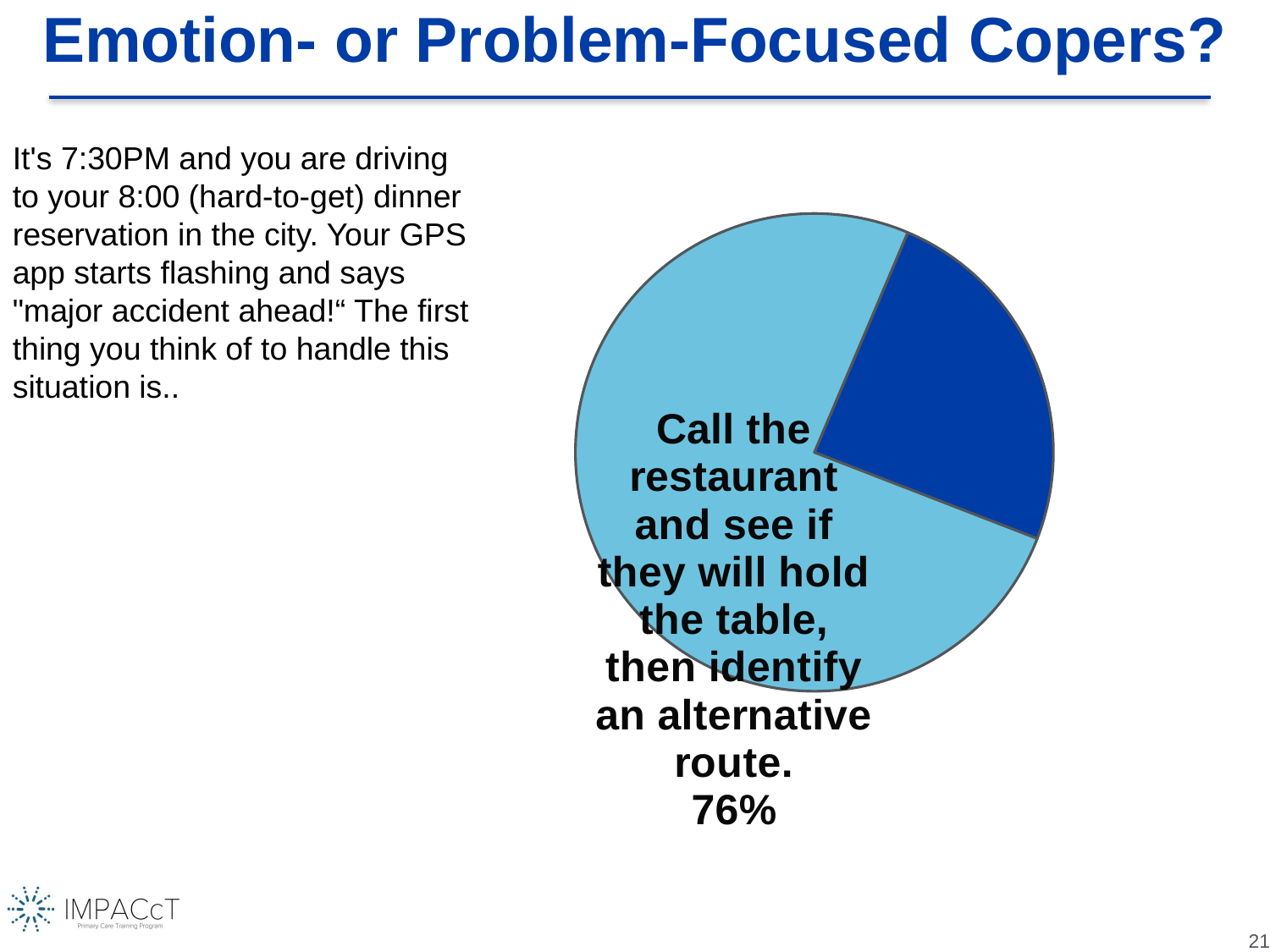

# Emotion- or Problem-Focused Copers?
It's 7:30PM and you are driving to your 8:00 (hard-to-get) dinner reservation in the city. Your GPS app starts flashing and says "major accident ahead!“ The first thing you think of to handle this situation is..
### Chart
| Category | Sales |
|---|---|
| Take deep breaths and tell yourself 'it's no big deal" | 12.0 |
| Call the restaurant and see if they will hold the table, then identify an alternative route. | 37.0 |
21

## Slide 22
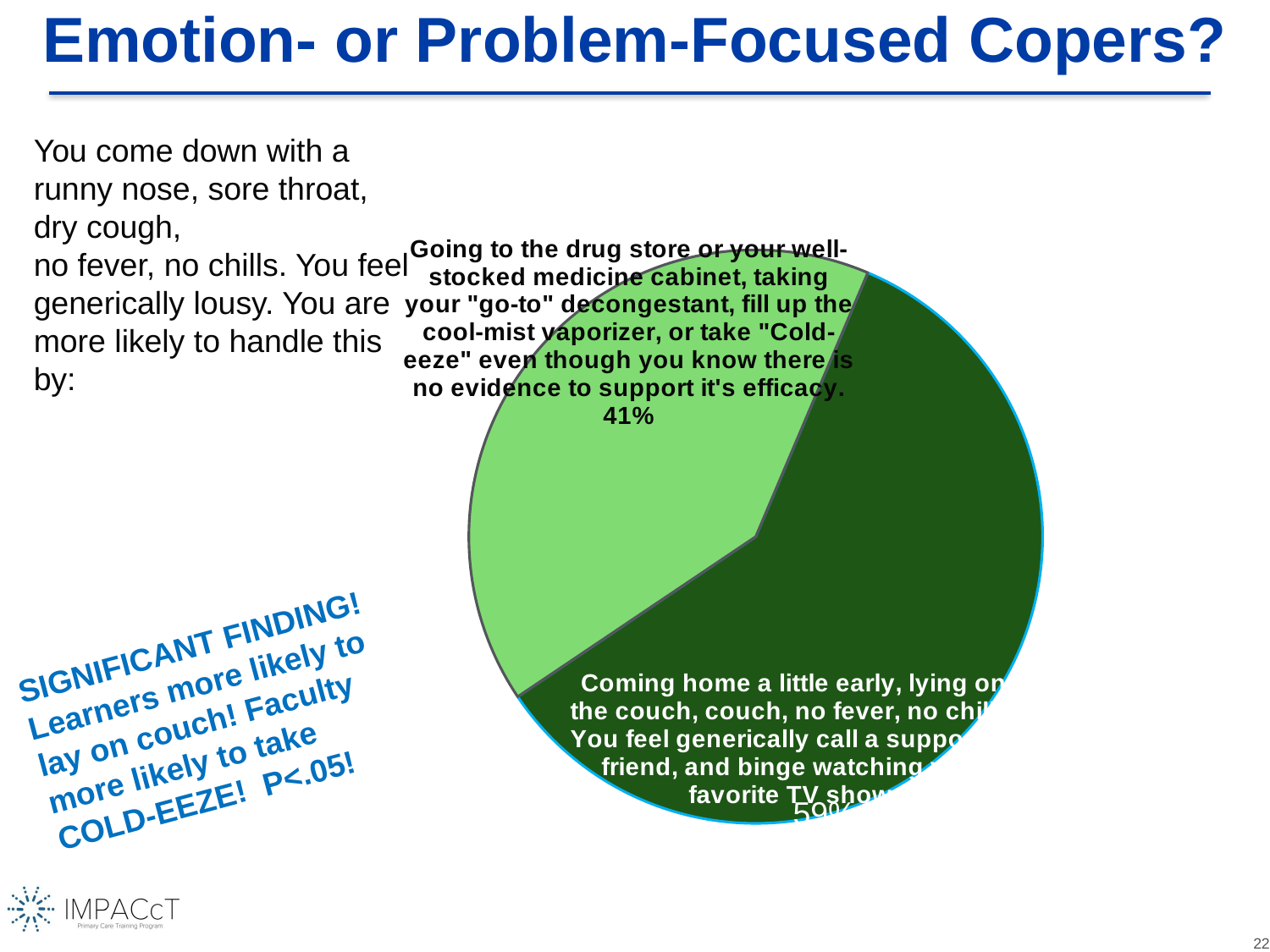

# Emotion- or Problem-Focused Copers?
### Chart
| Category | Sales |
|---|---|
| Coming home a little early, lying on the couch, couch, no fever, no chills. You feel generically call a supportive friend, and binge watching your favorite TV show. | 29.0 |
| Going to the drug store or your well-stocked medicine cabinet, taking your "go-to" decongestant, fill up the cool-mist vaporizer, or take "Cold-eeze" even though you know there is no evidence to support it's efficacy. | 20.0 |You come down with a runny nose, sore throat, dry cough,
no fever, no chills. You feel generically lousy. You are more likely to handle this by:
SIGNIFICANT FINDING!
Learners more likely to lay on couch! Faculty more likely to take COLD-EEZE! P<.05!
59%
22

## Slide 23
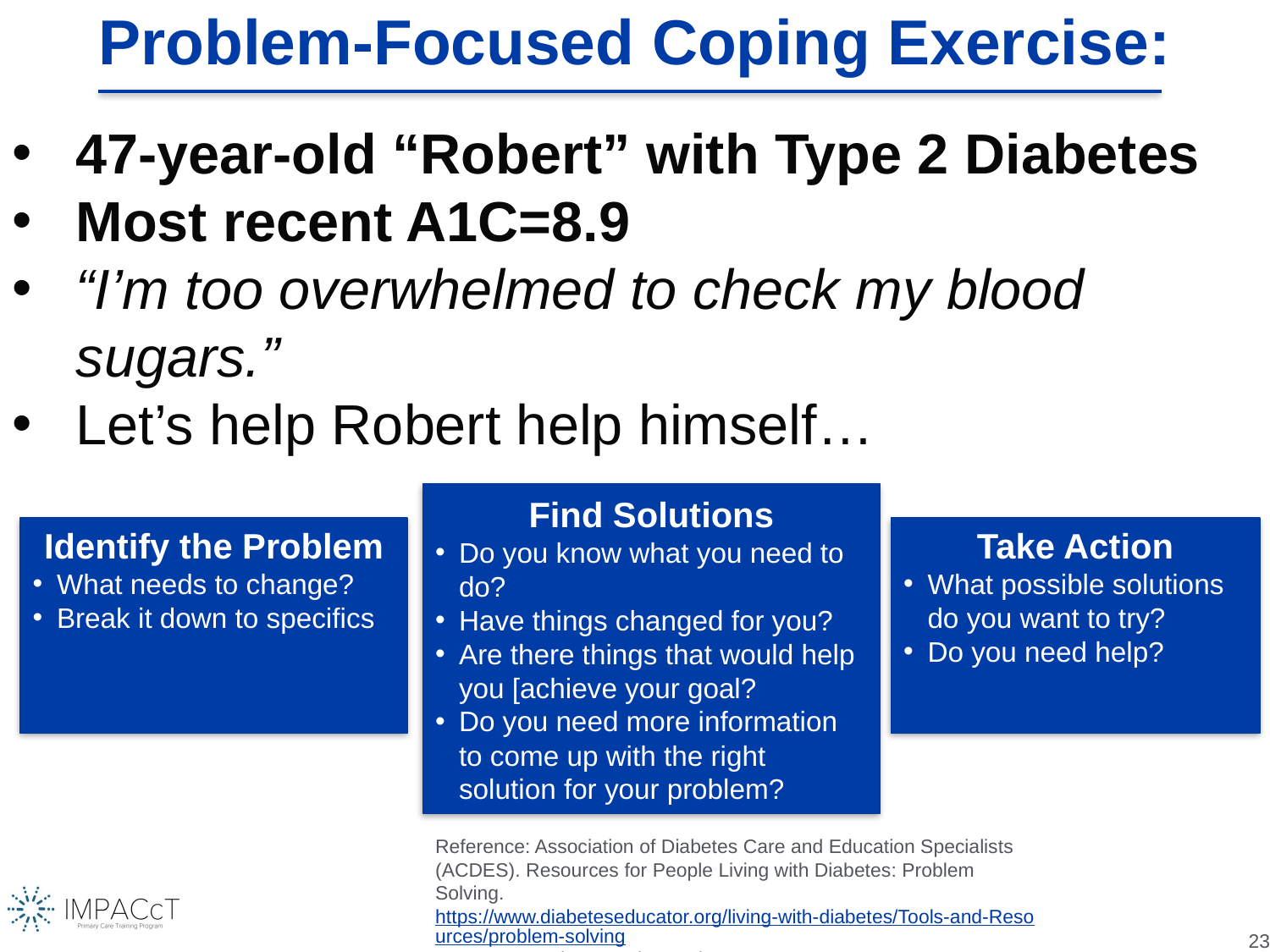

# Problem-Focused Coping Exercise:
47-year-old “Robert” with Type 2 Diabetes
Most recent A1C=8.9
“I’m too overwhelmed to check my blood sugars.”
Let’s help Robert help himself…
Find Solutions
Do you know what you need to do?
Have things changed for you?
Are there things that would help you [achieve your goal?
Do you need more information to come up with the right solution for your problem?
Identify the Problem
What needs to change?
Break it down to specifics
Take Action
What possible solutions do you want to try?
Do you need help?
Reference: Association of Diabetes Care and Education Specialists (ACDES). Resources for People Living with Diabetes: Problem Solving. https://www.diabeteseducator.org/living-with-diabetes/Tools-and-Resources/problem-solving Last accessed September 27th, 2021
23

## Slide 24
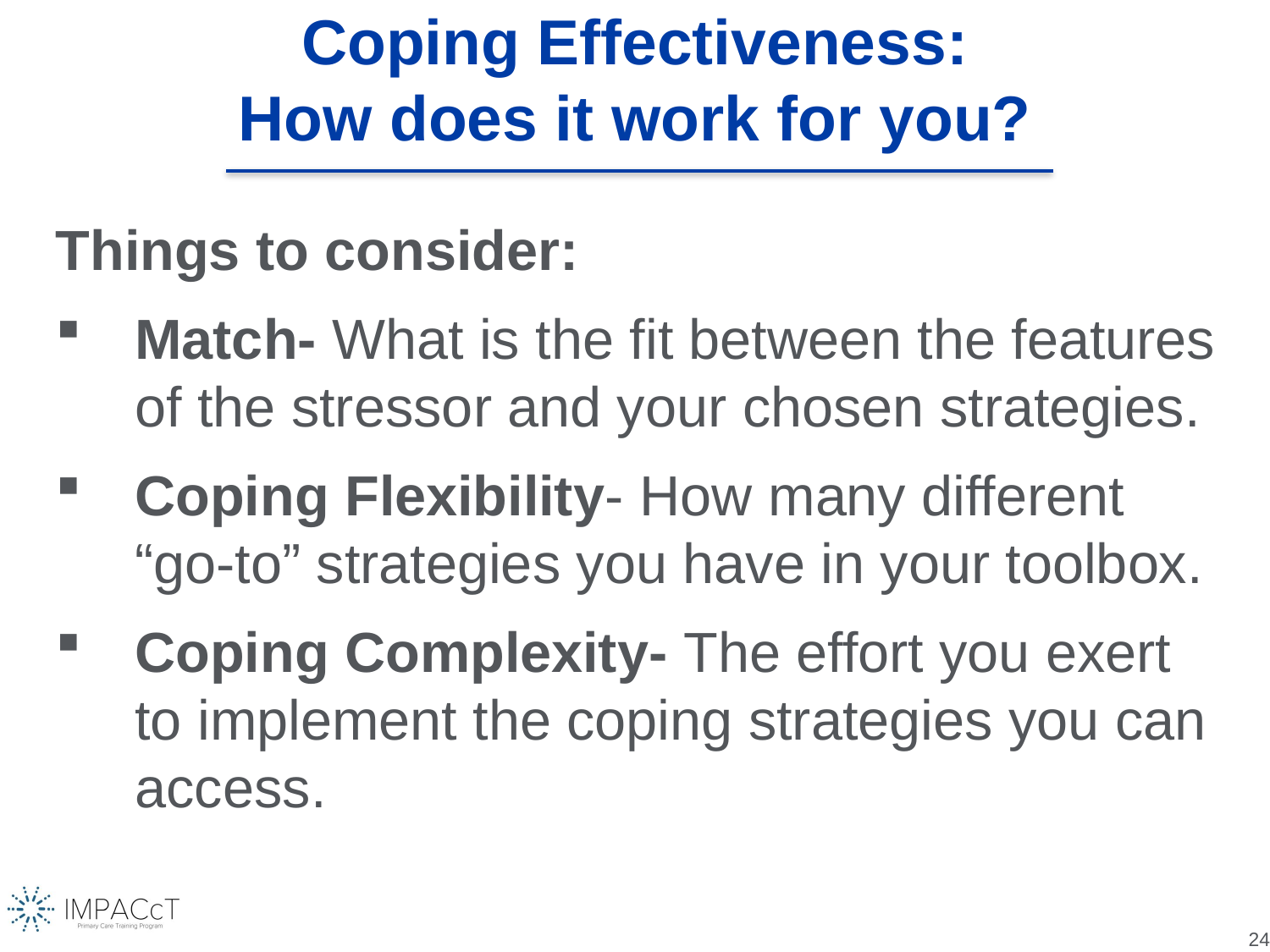

# Coping Effectiveness: How does it work for you?
Things to consider:
Match- What is the fit between the features of the stressor and your chosen strategies.
Coping Flexibility- How many different “go-to” strategies you have in your toolbox.
Coping Complexity- The effort you exert to implement the coping strategies you can access.
24

## Slide 25
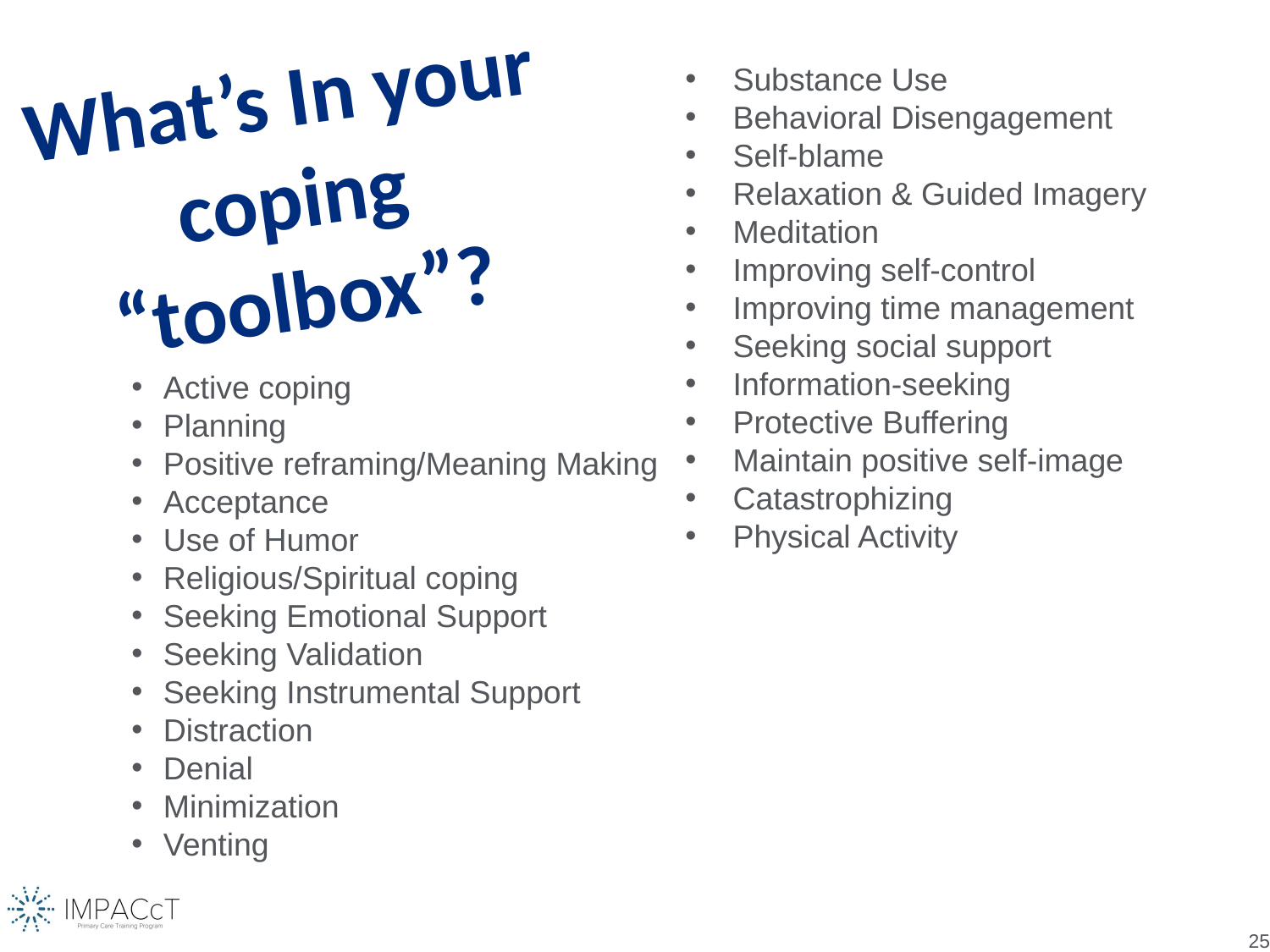

What’s In your coping “toolbox”?
Substance Use
Behavioral Disengagement
Self-blame
Relaxation & Guided Imagery
Meditation
Improving self-control
Improving time management
Seeking social support
Information-seeking
Protective Buffering
Maintain positive self-image
Catastrophizing
Physical Activity
Active coping
Planning
Positive reframing/Meaning Making
Acceptance
Use of Humor
Religious/Spiritual coping
Seeking Emotional Support
Seeking Validation
Seeking Instrumental Support
Distraction
Denial
Minimization
Venting
25

## Slide 26
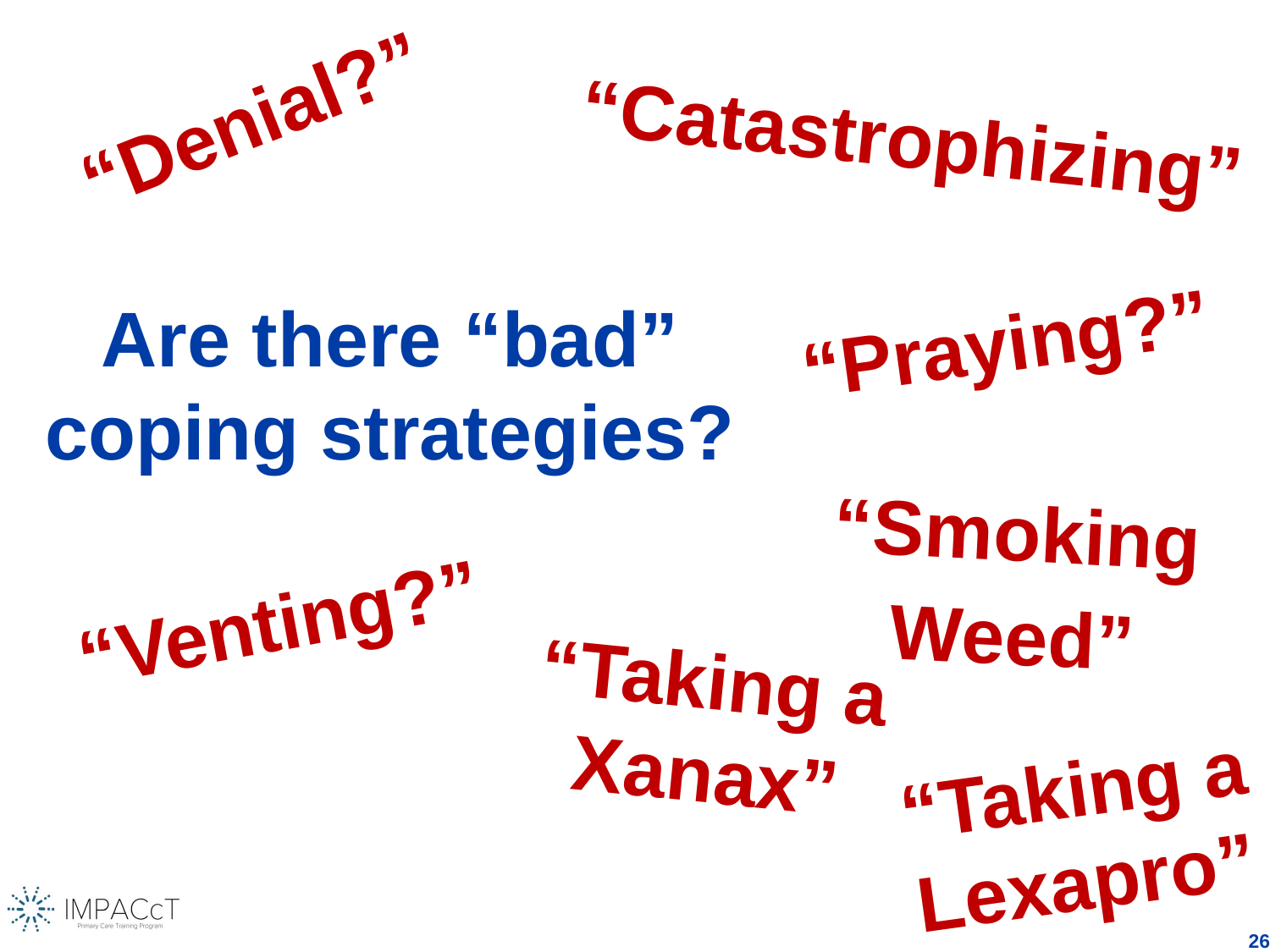

“Denial?”
“Catastrophizing”
Are there “bad” coping strategies?
“Praying?”
“Smoking
Weed”
“Venting?”
“Taking a Xanax”
“Taking a Lexapro”
26

## Slide 27
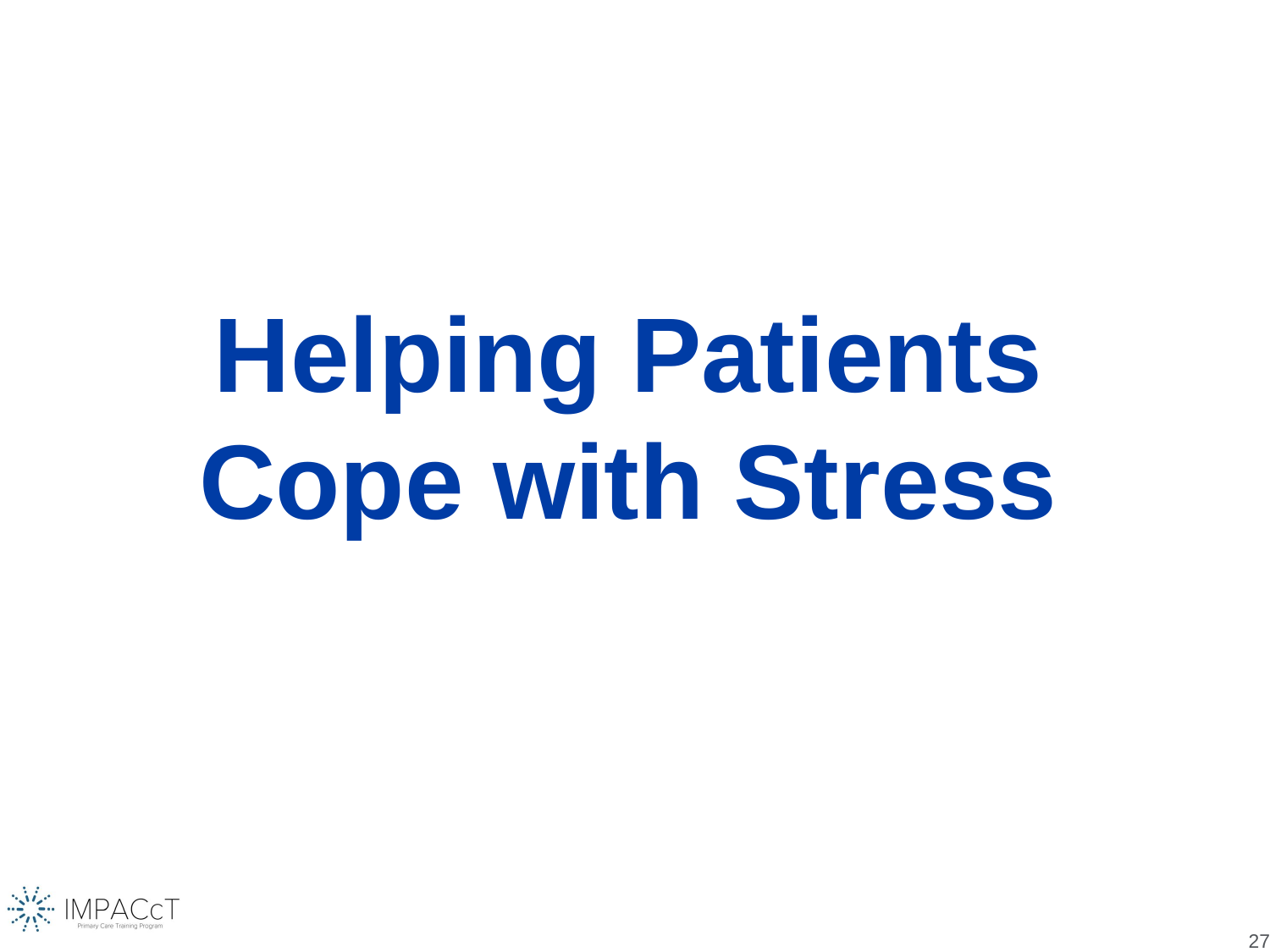

# Helping Patients Cope with Stress
27

## Slide 28
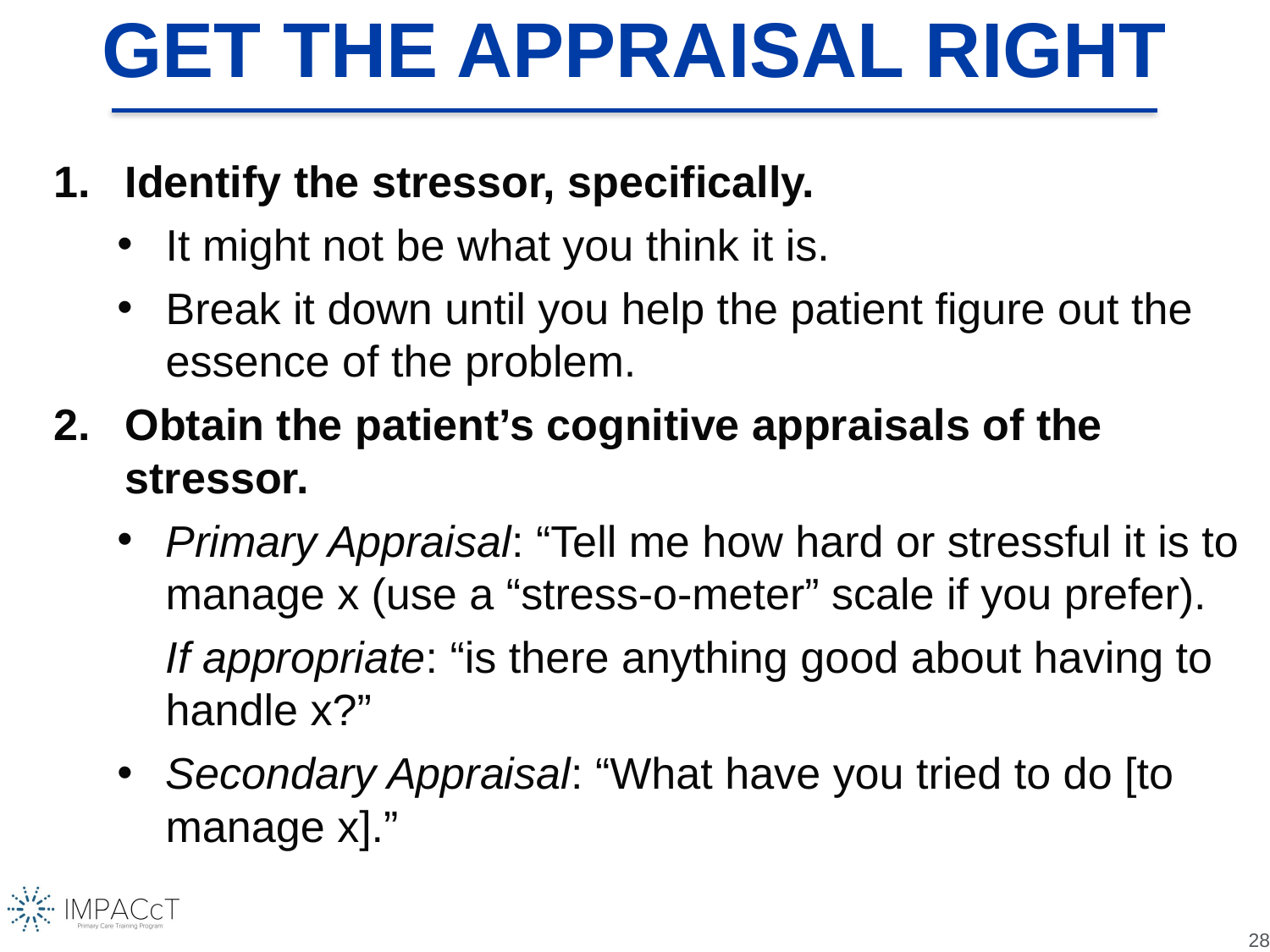

# GET THE APPRAISAL RIGHT
Identify the stressor, specifically.
It might not be what you think it is.
Break it down until you help the patient figure out the essence of the problem.
Obtain the patient’s cognitive appraisals of the stressor.
Primary Appraisal: “Tell me how hard or stressful it is to manage x (use a “stress-o-meter” scale if you prefer).
If appropriate: “is there anything good about having to handle x?”
Secondary Appraisal: “What have you tried to do [to manage x].”
28

## Slide 29
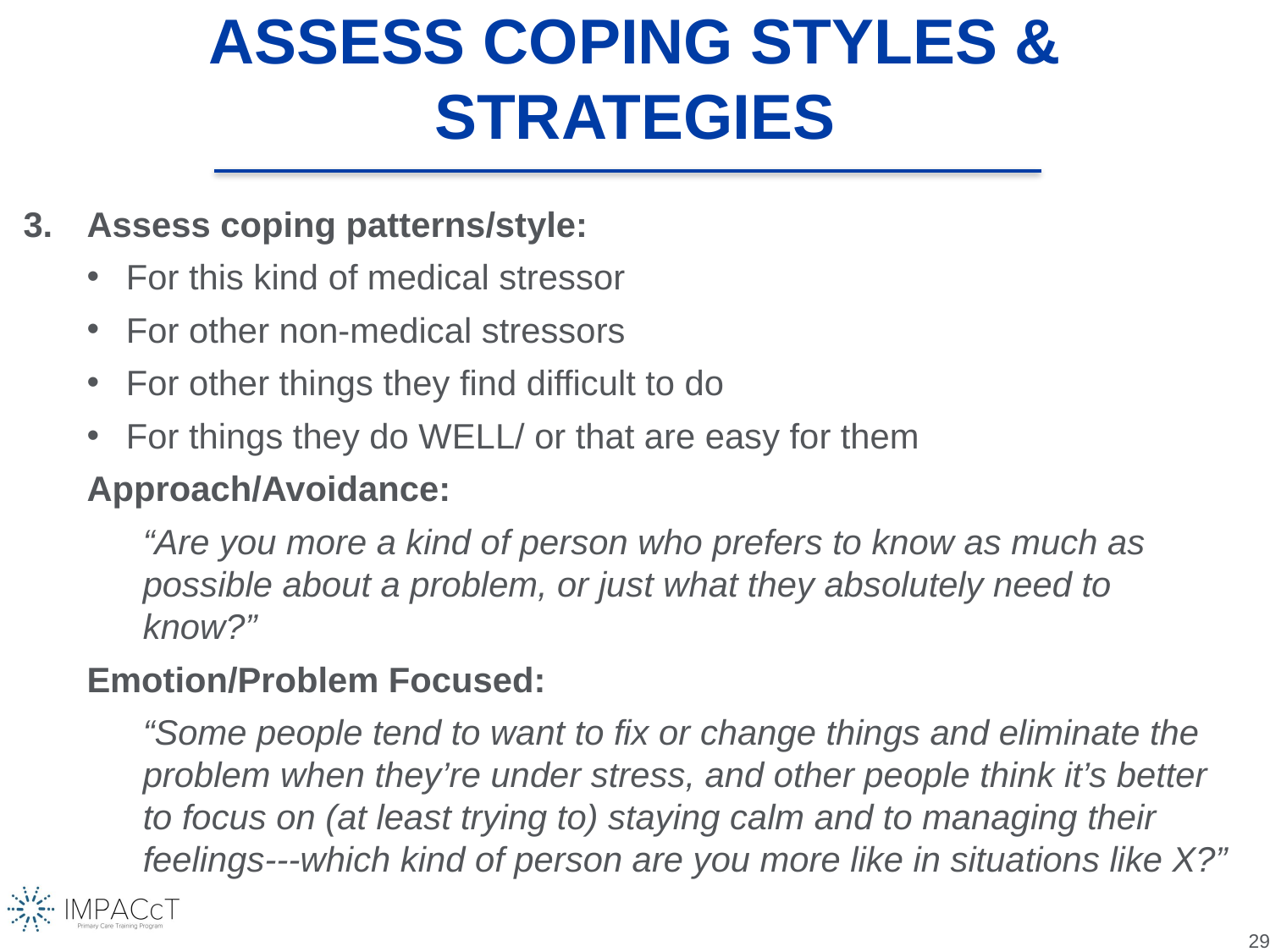

# ASSESS COPING STYLES & STRATEGIES
Assess coping patterns/style:
For this kind of medical stressor
For other non-medical stressors
For other things they find difficult to do
For things they do WELL/ or that are easy for them
Approach/Avoidance:
“Are you more a kind of person who prefers to know as much as possible about a problem, or just what they absolutely need to know?”
Emotion/Problem Focused:
“Some people tend to want to fix or change things and eliminate the problem when they’re under stress, and other people think it’s better to focus on (at least trying to) staying calm and to managing their feelings---which kind of person are you more like in situations like X?”
29

## Slide 30
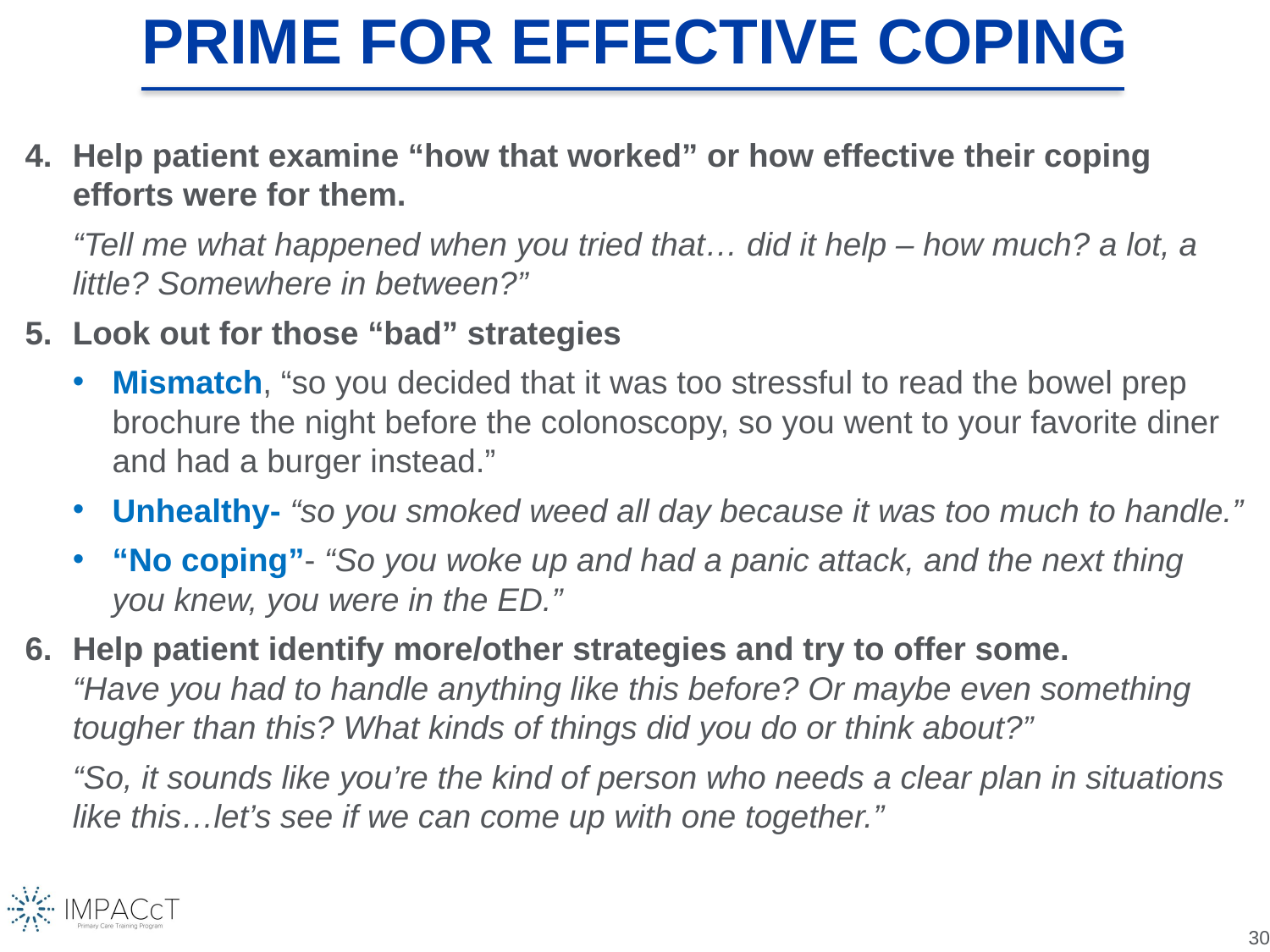

# PRIME FOR EFFECTIVE COPING
Help patient examine “how that worked” or how effective their coping efforts were for them.
“Tell me what happened when you tried that… did it help – how much? a lot, a little? Somewhere in between?”
Look out for those “bad” strategies
Mismatch, “so you decided that it was too stressful to read the bowel prep brochure the night before the colonoscopy, so you went to your favorite diner and had a burger instead.”
Unhealthy- “so you smoked weed all day because it was too much to handle.”
“No coping”- “So you woke up and had a panic attack, and the next thing you knew, you were in the ED.”
Help patient identify more/other strategies and try to offer some.
“Have you had to handle anything like this before? Or maybe even something tougher than this? What kinds of things did you do or think about?”
“So, it sounds like you’re the kind of person who needs a clear plan in situations like this…let’s see if we can come up with one together.”
30

## Slide 31
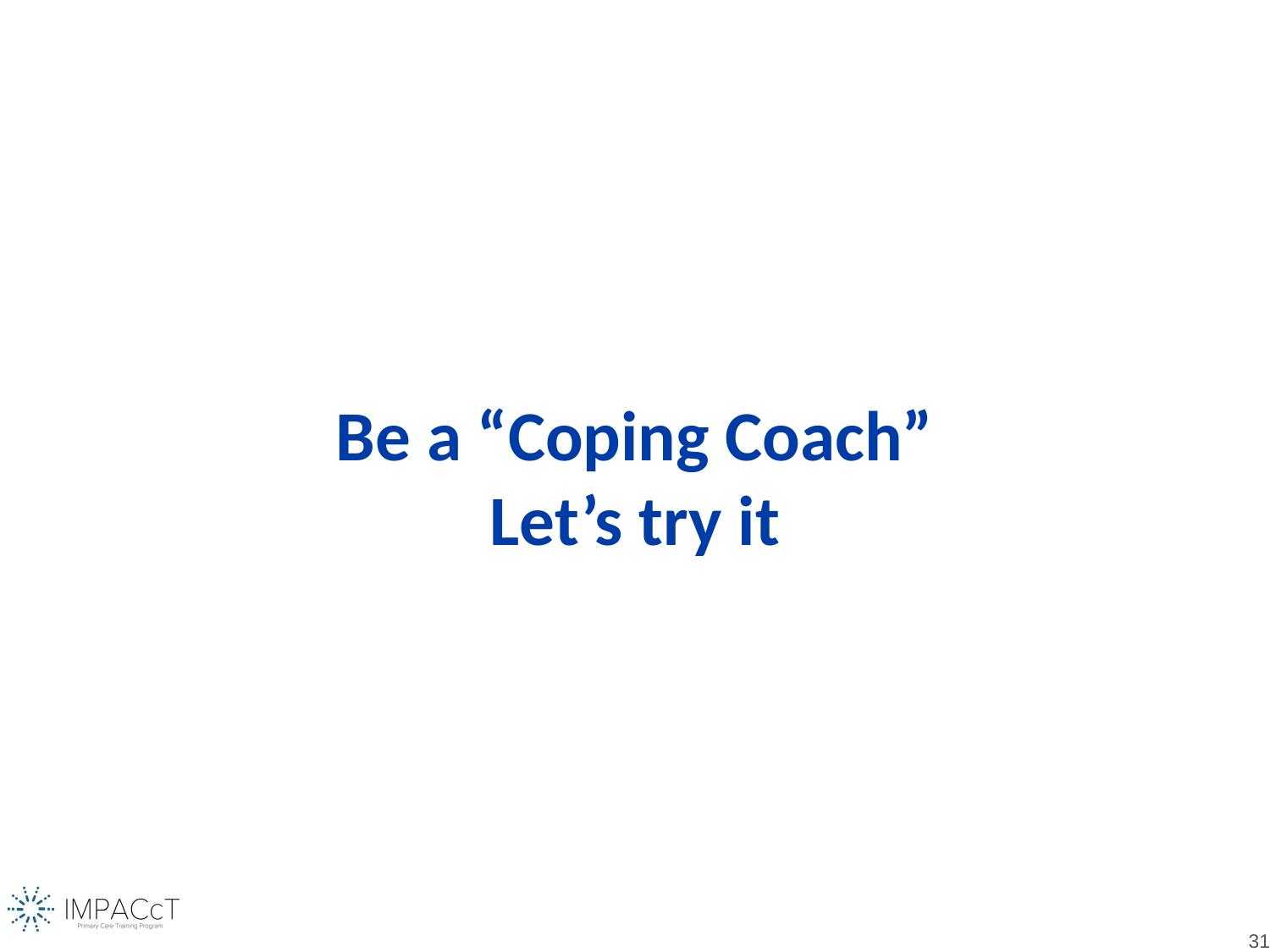

Be a “Coping Coach”
Let’s try it
31

## Slide 32
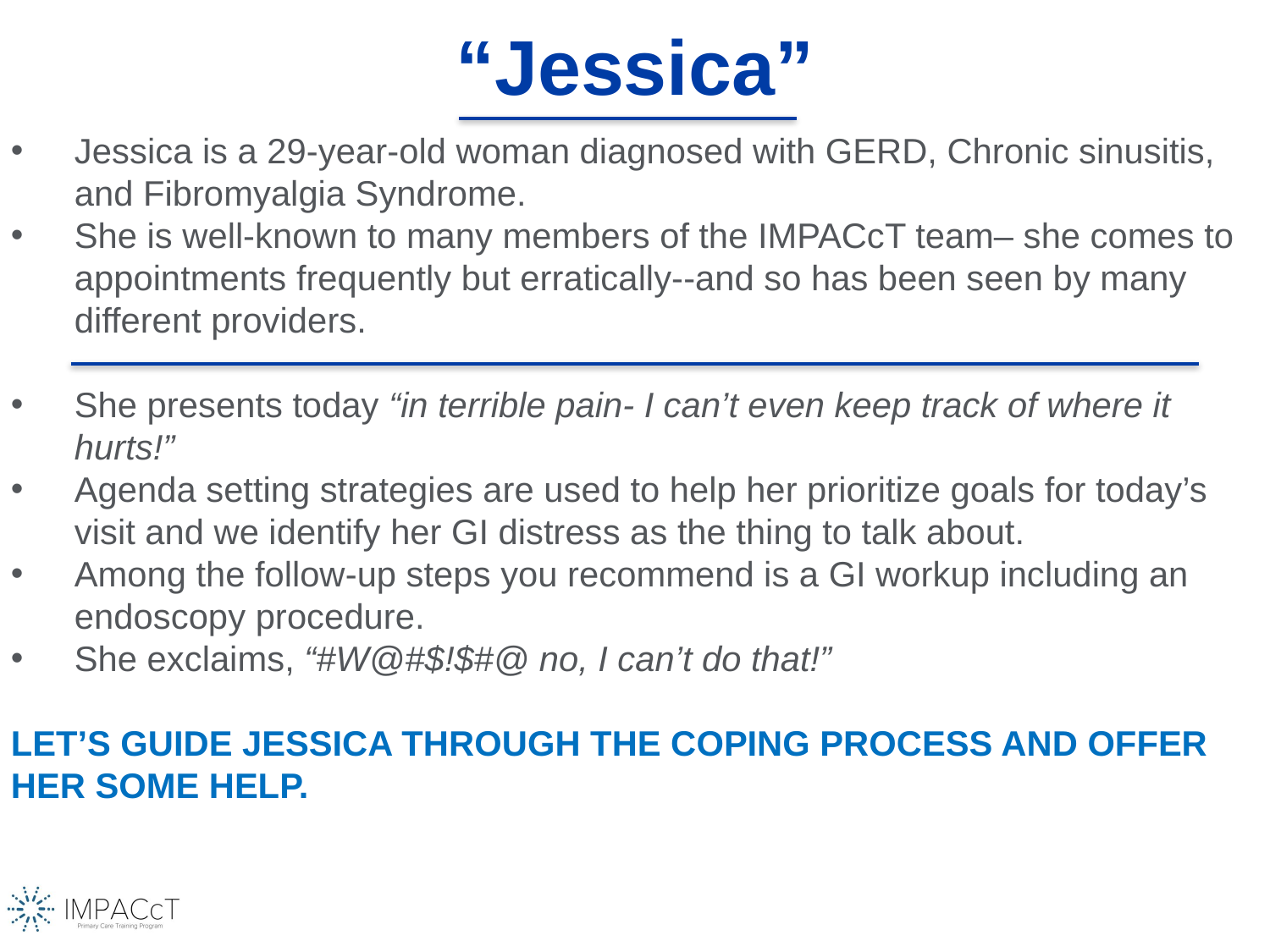

# “Jessica”
Jessica is a 29-year-old woman diagnosed with GERD, Chronic sinusitis, and Fibromyalgia Syndrome.
She is well-known to many members of the IMPACcT team– she comes to appointments frequently but erratically--and so has been seen by many different providers.
She presents today “in terrible pain- I can’t even keep track of where it hurts!”
Agenda setting strategies are used to help her prioritize goals for today’s visit and we identify her GI distress as the thing to talk about.
Among the follow-up steps you recommend is a GI workup including an endoscopy procedure.
She exclaims, “#W@#$!$#@ no, I can’t do that!”
LET’S GUIDE JESSICA THROUGH THE COPING PROCESS AND OFFER HER SOME HELP.
32

## Slide 33
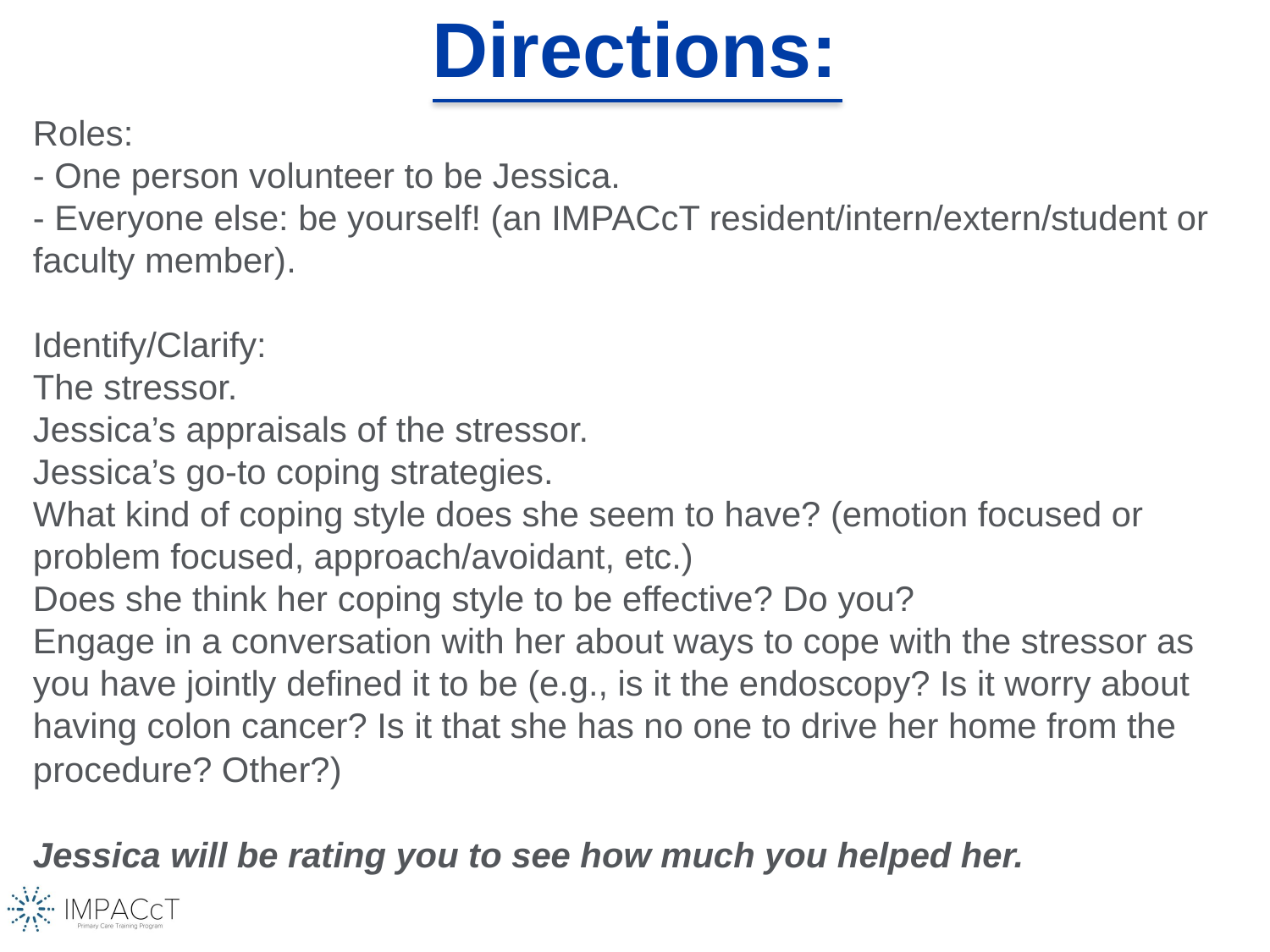

# Directions:
Roles:
- One person volunteer to be Jessica.
- Everyone else: be yourself! (an IMPACcT resident/intern/extern/student or faculty member).
Identify/Clarify:
The stressor.
Jessica’s appraisals of the stressor.
Jessica’s go-to coping strategies.
What kind of coping style does she seem to have? (emotion focused or problem focused, approach/avoidant, etc.)
Does she think her coping style to be effective? Do you?
Engage in a conversation with her about ways to cope with the stressor as you have jointly defined it to be (e.g., is it the endoscopy? Is it worry about having colon cancer? Is it that she has no one to drive her home from the procedure? Other?)
Jessica will be rating you to see how much you helped her.
33

## Slide 34
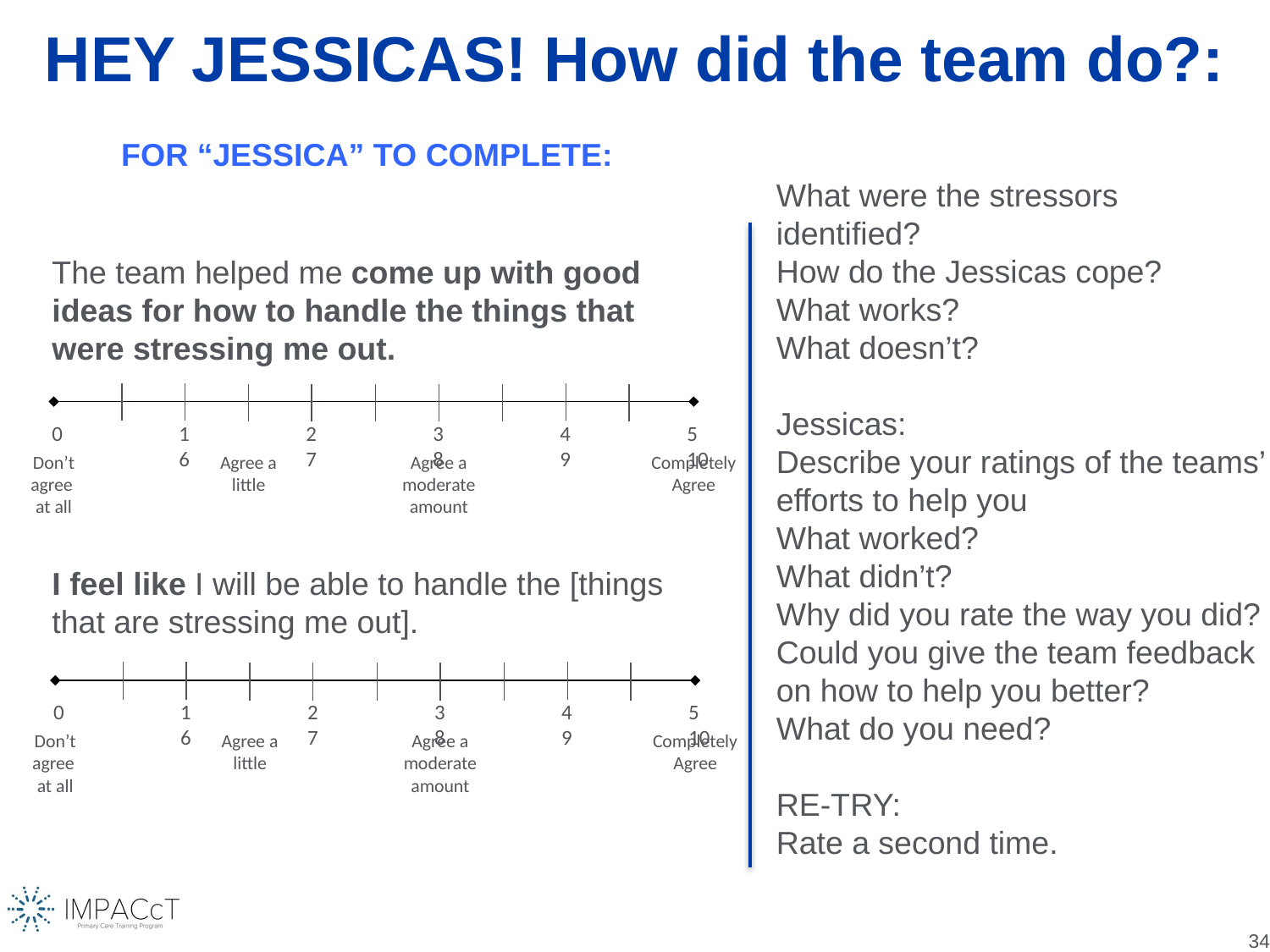

# HEY JESSICAS! How did the team do?:
FOR “JESSICA” TO COMPLETE:
What were the stressors identified?
How do the Jessicas cope?
What works?
What doesn’t?
Jessicas:
Describe your ratings of the teams’ efforts to help you
What worked?
What didn’t?
Why did you rate the way you did?
Could you give the team feedback on how to help you better?
What do you need?
RE-TRY:
Rate a second time.
The team helped me come up with good ideas for how to handle the things that were stressing me out.
0	1	2	3	4	5	6	7	8	9	10
Agree a little
Agree a moderate amount
Don’t agree
at all
Completely Agree
I feel like I will be able to handle the [things that are stressing me out].
0	1	2	3	4	5	6	7	8	9	10
Agree a little
Agree a moderate amount
Don’t agree
at all
Completely Agree
34

## Slide 35
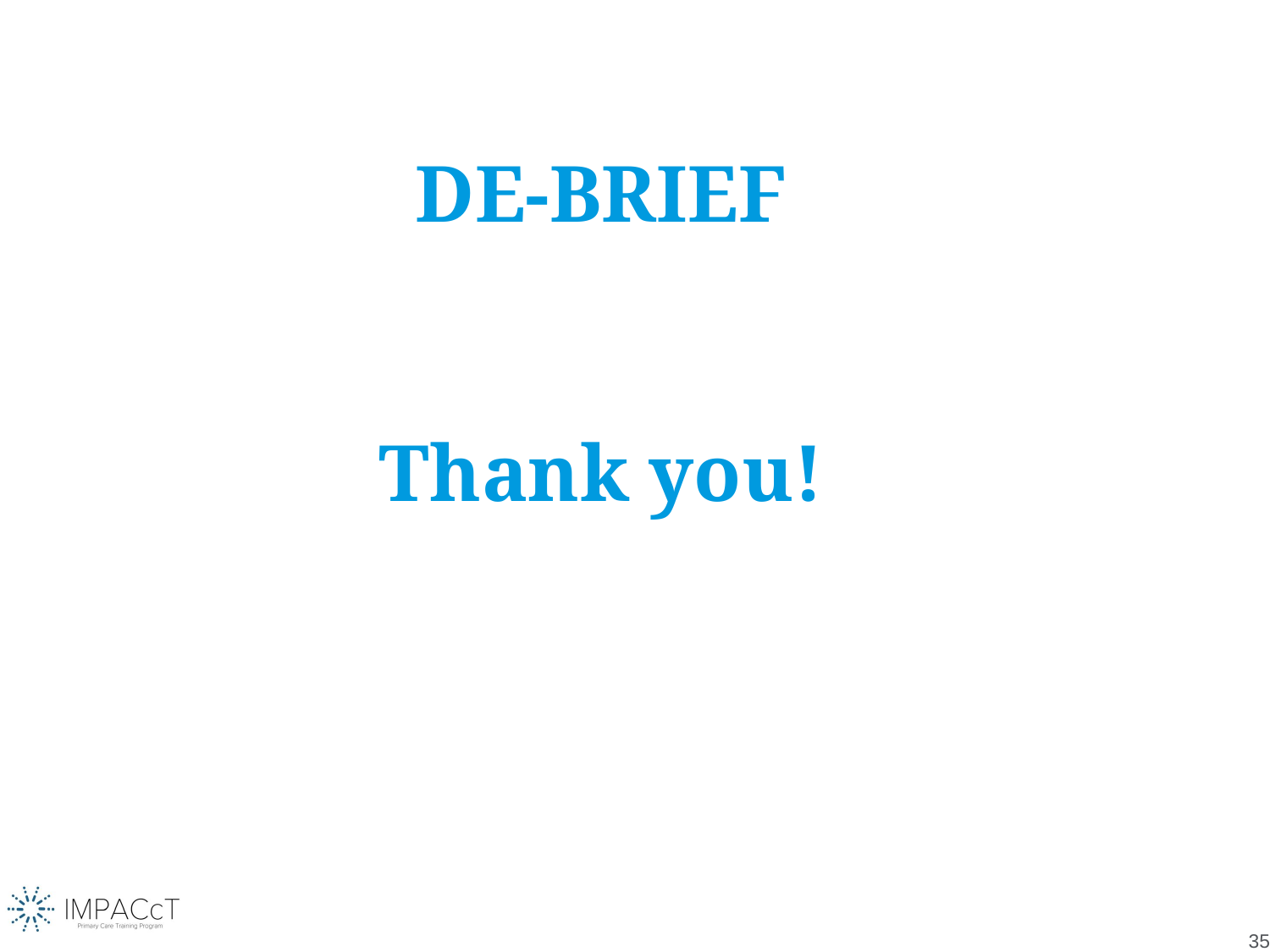

# DE-BRIEF Thank you!
35
