# Supplementary material for: Everybody Copes: An Interprofessional Workshop on Stress, Coping, and Helping Primary Care Patients Manage Medical Stressors
Source: MedEdPORTAL. 2023 Feb 14;19:11300. doi: 10.15766/mep_2374-8265.11300 (PMC9925639; doi:10.15766/mep_2374-8265.11300)
Supplement: Supplementary file 1 — Prework.docxSlide Presentation.pptxMindfulness Script.docxEvaluation.docx [file mep_2374-8265.11300-s001.zip › C. Mindfulness Script.docx]

**Appendix C: Mindful Eating Script**

Begin by connecting to your breath and body. Notice the sensations you feel in your chair. Don’t try to change anything, just notice what it feels like.

Feel yourself sitting with your body in your chair, feel your feet on the ground. Notice where your hands and arms are situated.

With your awareness in this moment, notice any thoughts, sensations or emotions you are experiencing. Tune into the awareness or sensation that you have in your body of feeling hungry, thirsty or maybe even feeling full. Just pay attention to the sensations that give you this information.

Now pick up the hershey’s kiss in front of you, holding it between your thumb and finger.

Imagine it were a novel item, and you’ve never seen a Hershey’s kiss before in your life.

Notice how it feels in your hand, its weight, the temperature, is it smooth or bumpy.

Slowly, unwrap the chocolate.

Notice how your fingers move together to unwrap it. How does the foil feel in your fingers? How about the chocolate?

Observe with curiosity as you pay attention to the shape, any grooves, and surfaces. Is it soft or hard? Notice any thoughts you might have about chocolate - any memories about chocolate, or feelings of liking or disliking it.

Now, take the piece of chocolate and bring it toward your nose.

Slowly inhale, and try to smell the chocolate with your full awareness.

What does it smell like? Perhaps it’s difficult for you to smell at this time, and that’s ok. Just notice that experience of smelling, however it feels for you.

Take a moment to smell it again. Notice if you have any memories, sensations or reactions anywhere in your body. In your mouth, your salivary glands, any other systems in your body.

Slowly move the chocolate towards your mouth, paying attention to all of the coordination involved in that movement.

With the chocolate close to your mouth, place it on your tongue, without biting into it. Pay attention to how it tastes. What are the flavors that you notice? Does it taste the same as it smelled? Notice any reactions you have, or any memories you have associated with this taste. How is your mouth responding?

Without chewing or swallowing, experience what it’s like to bite into the chocolate. Notice how it feels on your teeth, and how it feels on your tongue. Notice the flavor, and the texture. Maybe consider rolling the chocolate around in your mouth, noticing any changes.

When you are ready, start to chew on your bite of chocolate.

Take time to chew without swallowing, noticing any changes over time. Pay attention to the coordinated movements, and how they impact your experience of chewing.

Notice any sounds you hear as you chew.

When you feel that you are finished chewing, swallow the chocolate.

Notice the path the chocolate takes as it moves from mouth and tongue, to throat, to esophagus, and all the way down to your stomach. Pay attention to each of these steps, any sensations as the chocolate moves through your digestive tract.

Perhaps you have some gratitude for this action, for your ability to swallow food. For your body’s ability to eat, and perhaps to enjoy, while protecting yourself from any harm of choking.

Once you have swallowed, notice any lingering sensation or taste in your mouth. How does your body feel now that you’ve swallowed? What is your mind’s reaction to taking in this piece of chocolate?

Slowly take a breath, and notice how your breathing impacts any taste, flavor, or experience of having had the chocolate in your mouth.

Now, as you take another slow breath, consider your reactions to this exercise. Without judging, without judging yourself, just notice how you feel. Perhaps try taking another bite, again paying attention to the full experience, each component of eating. Take a few deep breaths, and notice how your body feels sitting in your chair again. Spend a moment with yourself in this experience, breathing, or eating mindfully, and in a moment or two we will wrap up.
