# Supplementary material for: Everybody Copes: An Interprofessional Workshop on Stress, Coping, and Helping Primary Care Patients Manage Medical Stressors
Source: MedEdPORTAL. 2023 Feb 14;19:11300. doi: 10.15766/mep_2374-8265.11300 (PMC9925639; doi:10.15766/mep_2374-8265.11300)
Supplement: Supplementary file 1 — Prework.docxSlide Presentation.pptxMindfulness Script.docxEvaluation.docx [file mep_2374-8265.11300-s001.zip › D. Evaluation.docx]

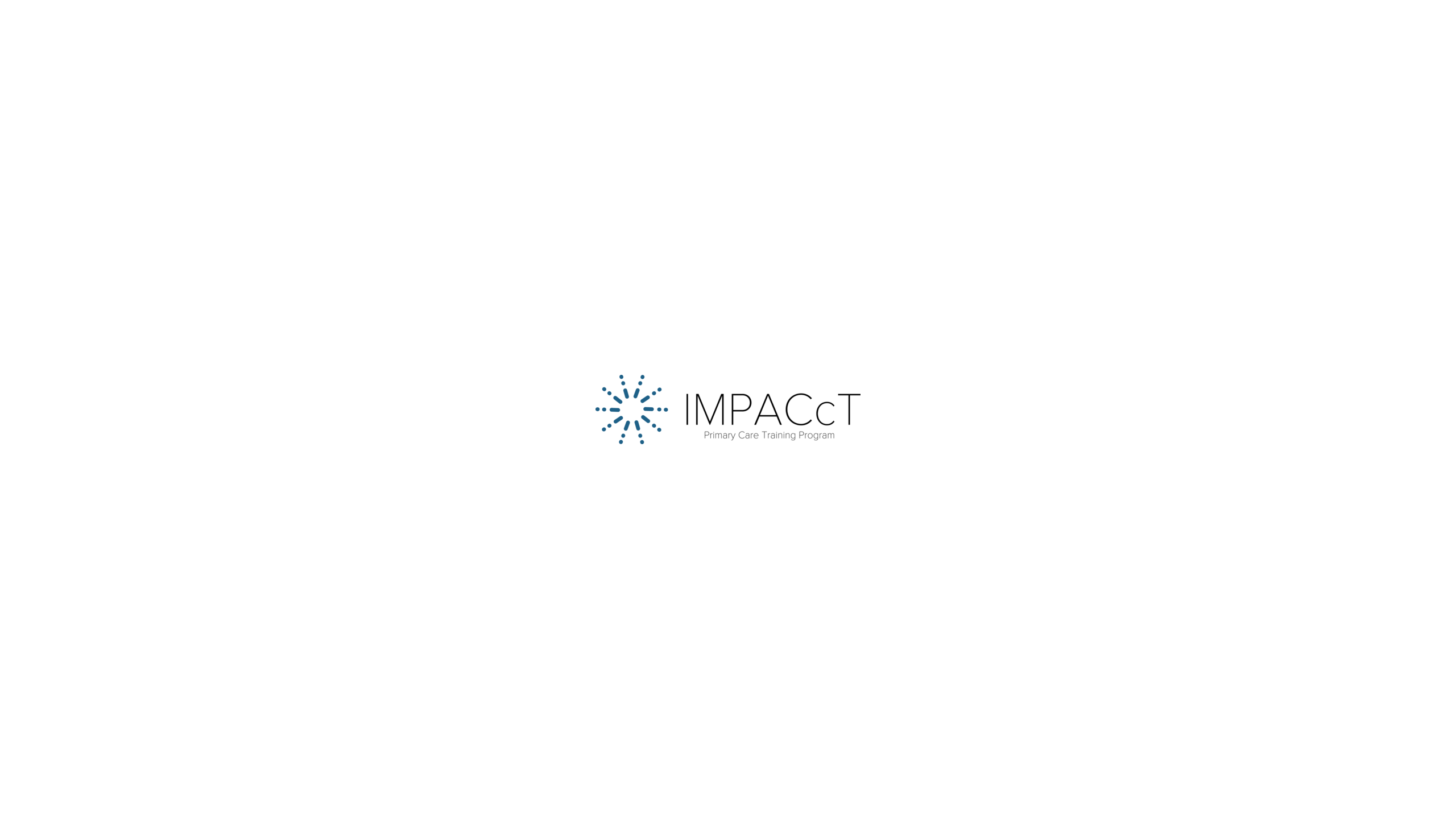


**I am a (check one):**

🞏 Medical Resident

🞏 Pharmacy Resident

🞏 Pharmacy Student

🞏 Psychology Extern

🞏 Medical Student

🞏 PA Student

🞏 Faculty/Attending 🞏 Support Team

🞏 Other (_____________)

**Everybody Copes: Understanding Ways to Manage Stress and**

**Helping Patients Cope Effectively with Medical Stressors**

**Program Evaluation**

Write down your “take-home message” from this retreat that you can apply to clinical situations.

Describe a skill or strategy that you learned today and how it might be useful to you in your work:

Describe something covered today that is ***confusing*** to you or unclear:

Complete this sentence: It would be great if the next retreat had **MORE…**

Complete this sentence: It would be great if the next retreat had **LESS**…

General Comments or Feedback [any constructive feedback welcome!]:

AFTER PARTICIPATING IN TODAY’S RETREAT…

**I think this workshop *made me more prepared* to talk with patients about stress in my role on an interprofessional primary care team.**

0 1 2 3 4 5 6 7 8 9 10

Don’t agree Agree a Agree a moderate Completely

at all a little amount Agree

**This retreat helped me learn skills that I can apply in the “real world.”**

0 1 2 3 4 5 6 7 8 9 10

Don’t agree Agree a Agree a moderate Completely

at all a little amount Agree

**Thank you!**
