# Supplementary material for: Everybody Copes: An Interprofessional Workshop on Stress, Coping, and Helping Primary Care Patients Manage Medical Stressors
Source: MedEdPORTAL. 2023 Feb 14;19:11300. doi: 10.15766/mep_2374-8265.11300 (PMC9925639; doi:10.15766/mep_2374-8265.11300)
Supplement: Supplementary file 1 — Prework.docxSlide Presentation.pptxMindfulness Script.docxEvaluation.docx [file mep_2374-8265.11300-s001.zip › A. Prework.docx]

*
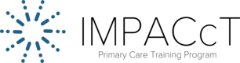
***IMPACcT Stress and Coping Inventory**

1) Today's date __________________________________ (mm-dd-yyyy)

2) I am a... (choose one)

□ Resident in Internal Medicine

□ Pharmacy /Intern/ Resident

□ Physician Assistant Student

□ Medical Student

□ FACULTY

□ Administrative Staff

People respond differently to stressful situations. For the following questions, please slide the bar to describe how challenging each situation is for YOU to manage, generally speaking.

**3) Getting stuck in traffic and realizing you'll be late for work/school.**

Not Stressful Moderately Extremely

At All Stressful Stressful

*(Place a mark on the scale above)*

**4) Going to a HUMONGOUS party at a friend of a friends...**

Not Stressful Moderately Extremely

At All Stressful Stressful

*(Place a mark on the scale above)*

**5) Being asked to see a patient with an "unusual” symptom presentation.**

Not Stressful Moderately Extremely

At All Stressful Stressful

*(Place a mark on the scale above)*

**6) Having bloodwork completed at your own annual Physical Exam.**

Not Stressful Moderately Extremely

At All Stressful Stressful

*(Place a mark on the scale above)*

**7) "Finals are next week!"- react!...**

Not Stressful Moderately Extremely

At All Stressful Stressful

*(Place a mark on the scale above)*

**8) Being asked to "deal with" a large spider that just crawled under the toaster oven in your kitchen.**

Not Stressful Moderately Extremely

At All Stressful Stressful

*(Place a mark on the scale above)*

**9) Walking into a crowded elevator.**

Not Stressful Moderately Extremely

At All Stressful Stressful

*(Place a mark on the scale above)*

**10) Seeing a patient who talks a lot.**

Not Stressful Moderately Extremely

At All Stressful Stressful

*(Place a mark on the scale above)*

**11) Going to the home of your husband's/wife's/significant other's family for a holiday dinner.**

Not Stressful Moderately Extremely

At All Stressful Stressful

*(Place a mark on the scale above)*

**12) Being asked to give a toast at your best friend's wedding.**

Not Stressful Moderately Extremely

At All Stressful Stressful

*(Place a mark on the scale above)*

**Part 2: Please choose one of the following options that BEST describes your response to the situation. YOU MUST CHOOSE ONE OR THE OTHER.**

**13) You are having bloodwork and the phlebotomist is getting ready to perform the venipuncture. Are you**

**more likely to....**

□ Look away, and stare at the clock on the wall--because you'd rather not know when it's going to happen.

□ Look right at the injection site or the needle--you need to know when this is going to happen.

**14) Your primary care provider tells you that they found a "shadow" on a chest x-ray. They said, “it’s probably nothing" However, they want you to schedule an MRI "out of an abundance of caution." Are you more likely to:**

□ Perform a PubMed literature search on lung cancer, followed by a different Google search every two until you have the MRI.

□ Try not to think about it until the day of the MRI appointment.

**15) It's 7:30PM and you are driving to your 8:00 (hard-to-get) dinner reservation in the city. Your Waze app starts flashing and says, "major accident ahead!” The first thing you think of to handle this situation is...**

□ Take deep breaths and say to yourself "it's no big deal."

□ Call the restaurant and see if they will hold the table, then identify an alternative route.

**16) You come down with a runny nose, sore throat, dry cough, no fever, no chills. You feel generically lousy. You are more likely to handle this by...**

□ Coming home a little early, lying on the sofa, calling a supportive friend, and binge watching your favorite TV show.

□ Going to the drug store or your well-stocked medicine cabinet, taking your "go-to" decongestant, fill up the cool-mist vaporizer, or take "Cold-eeze" even though you know there is no evidence to support its efficacy.

Thanks for your help-- we will present GROUP findings at our workshop next week. See you then!
